# Cholinergic Activation Enhances Resistance to Oral *Salmonella* Infection by Modulating Innate Immune Defense Mechanisms at the Intestinal Barrier

**DOI:** 10.3389/fimmu.2018.00551

**Published:** 2018-03-19

**Authors:** Ray M. Al-Barazie, Ghada Hassan Bashir, Mohammed M. Qureshi, Yassir A. Mohamed, Ashraf Al-Sbiei, Saeed Tariq, Wim J. Lammers, Basel K. al-Ramadi, Maria J. Fernandez-Cabezudo

**Affiliations:** ^1^Department of Biochemistry, College of Medicine and Health Sciences, United Arab University, Al-Ain, United Arab Emirates; ^2^Department of Medical Microbiology and Immunology, College of Medicine and Health Sciences, United Arab University, Al-Ain, United Arab Emirates; ^3^Department of Anatomy, College of Medicine and Health Sciences, United Arab University, Al-Ain, United Arab Emirates; ^4^Department of Physiology, College of Medicine and Health Sciences, United Arab University, Al-Ain, United Arab Emirates

**Keywords:** acetylcholine, cholinergic pathway, *Salmonella* infection, mucosal innate immunity, antimicrobial peptides, Paneth cells

## Abstract

Inflammation is a crucial defense mechanism that protects the body from the devastating effects of invading pathogens. However, an unrestrained inflammatory reaction may result in systemic manifestations with dire consequences to the host. The extent of activation of the inflammatory response is tightly regulated through immunological and neural pathways. Previously, we demonstrated that cholinergic stimulation confers enhanced protection in experimental animals orally infected with virulent *Salmonella enterica* serovar Typhimurium. In this study, we investigated the mechanism by which this enhanced protection takes place. Cholinergic stimulation was induced by a 3-week pretreatment with paraoxon, a highly specific acetylcholinesterase (AChE) inhibitor. This treatment enhanced host survival following oral-route infection and this correlated with significantly reduced bacterial load in systemic target organs. Enhanced protection was not due to increased gut motility or rapid bacterial clearance from the gastrointestinal tract. Moreover, protection against bacterial infection was not evident when the animals were infected systemically, suggesting that acetylcholine-mediated protective effect was mostly confined to the gut mucosal tissue. *In vivo* imaging demonstrated a more localized infection and delay in bacterial dissemination into systemic organs in mice pretreated with paraoxon. Morphological analysis of the small intestine (ileum) showed that AChE inhibition induced the degranulation of goblet cells and Paneth cells, two specialized secretory cells involved in innate immunity. Our findings demonstrate a crucial pathway between neural and immune systems that acts at the mucosal interface to protect the host against oral pathogens.

## Introduction

Various animal models have elucidated direct interplay between immune and nervous systems in controlling inflammation. The physiological mechanism through which the vagus nerve controls the immune response to inflammation has been named the inflammatory reflex” (1, 2). In the inflammatory reflex, the presence of inflammatory molecules in the periphery stimulates the afferent vagus nerve that relays the information to the brain (3). The brain then responds through the efferent vagus nerve and α7 subunit of the nicotinic acetylcholine receptor (AChR) and inhibits production of pro-inflammatory cytokines by activated macrophages.

The gut is continuously exposed to a variety of pathogen and non-pathogen antigens of dietary and environmental origin. In order to protect the host against the entrance of pathogens, the gastrointestinal tract is equipped with an intact epithelial barrier and effective innate immune mechanisms able to quickly respond to any possible pathogen transposing the epithelium (4). At the extra-epithelial level, the intestinal wall is protected by mucus and antimicrobial peptides (AMPs) released by goblet cells (GC) and Paneth cells (PC), respectively. PC are present in the crypts of Lieberkuhn, in the distal part of the small intestine, and are rich in secretory granules containing microbicidal peptides and proteins such as lysozyme, phospholipase A, cryptdins, cryptdin-related sequence peptides (CRS), and angiogenin-4 (Ang-4). These factors contribute to intestinal innate immunity by bacterial sequestering and limiting pathogen penetration and dissemination (5, 6). Furthermore, immune cells (macrophages and DC) located between the epithelial cells of the mucosal barrier recognize trespassing pathogens as foreigners and produce inflammatory cytokines and chemokines to recruit immune cells to the site of injury (7).

The nervous system (enteric as well as central) regulates several important intestinal functions. The central nervous system controls intestinal motility, secretion, and vasoregulation through both sympathetic and parasympathetic branches of the autonomic nervous system (8). Moreover, nerve axons have been identified in the close proximity of intestinal immune cells. This suggests an interaction between the immune and nervous system with the potential of modulating the immune response in the intestine. Specifically, noradrenergic fibers from the sympathetic nervous system have been found in the proximity of DCs in the PPs (9), plasma cells and T cells (10). Noradrenaline has also been reported to modulate cytokine response in T cells (11) as well as B cell proliferation and immunoglobulin secretion (12, 13). On the other hand, the cholinergic parasympathetic vagus nerve is able to modulate the immune response through ACh receptors on T cells, B cells, macrophages, and dendritic cells (14). However, the vagus nerve does not innervate the intestine lamina propria itself. Instead, it makes contact with cholinergic neurons of the enteric nervous system (8), which have been reported to be abundant in this site and located in close proximity to lymphoid cells (15). Importantly, the existence of interactions between the vagus nerve and sympathetic ganglia has also been repeatedly implicated (14). GC located in the Lieberkuhn crypts also express ACh surface receptors and are able to respond to the presence of ACh (16). Interestingly, cholinergic mechanisms were shown to be involved in the stimulation of both, goblet and PC in the intestine resulting in the secretion of antibacterial products (17).

Cholinergic stimulation leads to an attenuation of inflammatory responses and has a protective role in different animal models of inflammation, including murine sepsis (18, 19), splanchnic artery occlusion shock model (20), acute kidney injury (21), obesity (22), collagen-induced arthritis (23), and diabetes type 1 (24). In live infection models, our group previously demonstrated that inhibition of the acetylcholinesterase (AChE) activity, the enzyme that hydrolyzes the neurotransmitter ACh, increased host resistance to an oral infection with *Salmonella enterica* serovar Typhimurium (*S. typhimurium*) (25). Moreover, activation of muscarinic AChRs was shown to exert a protective role in *Salmonella* and *Nippostrogylus brasilinesis* infections (26). In the present study, we characterize the underlying mechanisms responsible for cholinergic pathway-induced protection to oral *Salmonella* infection. Our findings show that improved survival of infected mice was primarily due to decreased bacterial loads in target organs and fecal pellets. This correlated with a lower activation state of systemic immune responses in spleen and mesenteric lymph nodes (MLNs). Further, we provide evidence that cholinergic pathway activation upregulates the expression of AMPs by intestinal epithelial cells. Finally, we demonstrate that cholinergic stimulation induces the degranulation of intestinal secretory cells, including Paneth and GC, in a *Salmonella*-independent manner. Our findings suggest that enhanced cholinergic pathway-induced protection against an oral infection by virulent *Salmonella* is primarily mediated by innate immune mechanisms acting at the level of the gastrointestinal barrier.

## Materials and Methods

### Experimental Animals

Male BALB/c mice (the Jackson Laboratory, Bar Harbor, ME, USA), aged 8–12 weeks, were bred in the animal facility at the College of Medicine and Health Science (CMHS), UAE University, housed in plastic cages with a controlled light and dark cycle of 12 h each at 24–26°C and received rodent chow and water *ad libitum*. All studies involving animals were carried out in accordance with, and after approval of the Animal Research Ethics Committee of the CMHS (Protocol # AE/06/81).

### Paraoxon Preparation

Paraoxon ethyl (Sigma Chemicals Co., St. Louis, MO, USA) stock solution was prepared at a concentration of 10 mmol/l in anhydrous acetone. Working solution was prepared *ex tempore* in PBS to a final concentration of 80 nmol/ml. Each mouse received 40 nmol/0.5 ml/day of paraoxon (equivalent to 0.44 mg/kg of body weight) by intraperitoneal (i.p.) injection. Control animals received an equivalent volume of PBS.

### Experimental Protocol

BALB/c mice were randomly assigned into two groups. Group I served as control and received daily injection of sterile saline. Group II received daily injection of 40 nmol of paraoxon. All injections were given i.p. daily for 5 days, followed by a 2-day rest, and this cycle was repeated for a total of 3 weeks. Mice were weighed weekly, at which time blood was collected and analyzed for AChE activity in red blood cells (RBC), as detailed previously (24). At the end of treatment, half of the animals in each group were sacrificed and the other half infected (routinely following the 2-day break) i.p. or by oral route. *Salmonella*-infected animals were then either sacrificed at specific time points, as indicated, or followed for survival.

### Bacterial Strains and Preparation

Different bacterial strains of *S. typhimurium* were used in this study: virulent wild-type *S. typhimurium* (SL1344), attenuated double auxotrophic strain aroA^−^/aroD^−^ mutant (BRD509E) and a bioluminescent transfectant of SL1344 (SL1344:*lux*) tagged by expression of the lux operon from *Photorhabdus luminescens* (27), which was generously provided by Dr. Christopher Contag (Stanford University School of Medicine, Stanford, CA, USA). The characteristics and preparation of log-phase bacterial suspensions have been described elsewhere (25, 28, 29). All experiments conducted using *Salmonella* strains were done following institutional biohazard regulations in a BSL2 laboratory.

### Bacterial Inoculation

For systemic infection, BRD509E or SL1344 bacterial strains were injected i.p. in 0.5 ml volume, at the indicated doses. For oral inoculation, SL1344 was administered in 200 μl/mouse following a previously detailed procedure (25). The bacterial dose was confirmed by colony-forming units (CFUs) plate counts.

### Determination of Bacterial Load in Target Organs and Peritoneal Cavity

Procedures for determination of CFUs have been described previously (30). Mice were sacrificed at different time points after oral or i.p. infection. Spleen, liver, and MLNs were aseptically removed and homogenized in 1–2 ml cold PBS. Aliquots at an appropriate dilution were streaked on *Salmonella*–*Shigella* (SS) agar plates with (for BRD509E) or without (for SL1344) ampicillin. For bacterial determination in the gastrointestinal tract, mice were inoculated with SL1344 orally and sacrificed 1–3 days postinfection. The small intestine was aseptically removed and luminal content collected, weighed, and processed for determination of bacterial CFUs. In separate studies, the ileum region of the small intestine was collected, homogenized, and processed for bacterial determination. In all studies, identical regions of the small intestine were collected from all mice. To determine the bacterial loads in the peritoneal cavity, peritoneal fluid was harvested following injection of 10 ml cold Ca^2+^/Mg^2+^-free saline. Fluid was spun down, and cell pellet suspended in distilled water. Aliquots or appropriate dilutions were plated on SS agar plates with or without ampicillin. CFUs counts were determined after overnight incubation at 37°C.

### Determination of Bacterial Load and IgA Estimation From Fecal Pellets

Detailed procedures for the determination of bacterial load and sIgA content in feces have been published (31). Clarified fecal homogenates were plated on SS agar plates with streptomycin for the pellets collected from SL1344-infected animals, or streptomycin and ampicillin for pellets collected from BRD509E-infected mice. After overnight incubation at 37°C, CFUs counts were determined. To quantify the presence of sIgA, fecal pellets were homogenized in extraction buffer [1% BSA, 30 mM EDTA, and protease inhibitors cocktail III (Sigma)], and supernatants were collected after centrifugation and stored at −20°C until assayed for total and *Salmonella*-specific sIgA antibody levels.

### Estimation of Fecal sIgA by ELISA

The quantification of total sIgA and *Salmonella*-specific sIgA in fecal extracts has been described (31, 32). MaxiSorp 96-well plates (Nunc, Roskilde, Denmark) were coated with goat anti-mouse Ig H + L [IgA, IgG, and IgM] antibody (Southern Biotech, Birmingham, AL, USA) and incubated overnight at 4°C for total IgA. For detection of anti-*Salmonella* IgA, plates were coated with heat-killed *Salmonella* (1 × 10^6^ CFUs/ml) and incubated overnight at room temperature. IgA was detected using biotin-conjugated anti-mouse IgA antibody (Southern Biotech) followed by streptavidin-HRP. Color was developed by using TMB substrate solution and plate was read with the TECAN microplate reader (Maennedorf, Switzerland) at λ = 450 nm.

### Spleen and Peritoneal Exudate Cells (PECs) Preparation

Erythrocytes-depleted splenocytes and isolated PECs were prepared in supplemented RPMI-1640 medium with 5% fetal calf serum (all purchased from Hyclone, UT, USA), as previously described (32). Splenocytes (5 × 10^6^ or 3 × 10^6^ cells/ml) and PECs (1.5 × 10^6^ cells/0.5 ml) were cultured for 24 or 48 h at 37°C in 5% CO_2_. Culture supernatants were collected, spun free of any cells and kept at −20°C until assayed for cytokines and nitric oxide (NO) content.

### Cytokine Analysis and NO Determination

Supernatants of *ex vivo*-cultured cells and serum samples were analyzed for cytokine content as detailed previously (32), using BD OptEIA ELISA mouse kits (BD, Franklin Lakes, NJ, USA) according to manufacturer’s instructions. The sensitivity of detection was ~30 pg/ml for IFN-γ and IL-10 and ~15 pg/ml for IL-6 and IL-12/IL-23p40. Supernatants were also analyzed for NO production (33) by determine the accumulation of nitrite according to the Griess method. Nitrite concentration was determined from a standard curve prepared using sodium nitrite (5–100 µM), 5 µM being the minimum limit of detection.

### Purification of Spleen Myeloid Cells

Spleen cells were magnetically labeled using microbeads conjugated to monoclonal anti-mouse CD11b antibody (BD Biosciences, San Jose, CA, USA) and positively selected by magnetic separation using the autoMACS separator (Miltenyl Biotec, Bergisch Gladbach, Germany). Flowcytometric analysis of purified cells confirmed >90% purity.

### Phagocytosis Assay

Phagocytic capability of purified splenic CD11b^+^ cells was measured by Vybrant Phagocytosis Assay Kit (Invitrogen, Life Technologies, Carlsbad, CA, USA) which contains Fluorescein-labeled *Escherichia coli* K-12 BioParticles. Bioparticle suspension was prepared, following the manufacturer protocol, and added to purified CD11b^+^ spleen cells (1 × 10^5^/50 μl/well, in DMEM). After 2-h incubation, trypan blue was added to quench the extracellular fluorescence. Intracellular uptake was quantified by measuring fluorescence emitted by engulfed bacteria at 485 nm excitation and 535 nm emission using a Victor X3 2030 microplate reader (PerkinElmer, Waltham, MA, USA). Measurements of negative and positive controls as well as experimental samples were made in four replicates and their averages used for calculations. Results were expressed as fold increase from controls.

### Flow Cytometric Analysis

Erythrocytes-depleted spleen cells, MLN cells and PECs were analyzed using a 6-color FACS, following a standard protocol (34). Cells were stained with different combinations of conjugated monoclonal antibodies CD3-FITC (Biolegend, San Diego, CA, USA), CD19-PEcy7, CD8-APCcy7, Sca-1-PE, CD25-APC, CD69-PEcy7, CD86-PE (BD Biosciences), CD11b-APCcy7, CD11c-APC, CD4-PE, F4/80-PEcy7, MHC II-APC (eBioscience, San Diego, CA, USA), and CD40-FITC (Southern Biotech). In the first panel, cells were immunphenotyped using mAbs to CD3, CD19, CD11b, CD11c, and Sca-1. A second panel was used to analyze T cell subsets and their activation status and consisted of mAbs to CD3, CD4, CD8, CD25, and CD69. In the third panel, myeloid cell activation status was analyzed using mAbs to CD11b, F4/80, CD40, CD86, and MHC class II. Non-viable cells that stained positive for 7-AAD (eBioscience), were excluded from the analysis. For each antibody, appropriate isotype control was used. Data were collected on 30,000 cells using BD FACS Canto II (BD Biosciences) and analyzed using BD FACSDiva software (BD).

### Intestinal Transit

At day 2 posttreatment with paraoxon or saline, mice were fasted overnight. The following day, mice received 100 µl of 5% Evans blue suspension by oral gavage and sacrificed 15 min later. The small intestine was removed and spread on a flat surface. The distance traveled by the Evans blue dye was measured in centimeters and calculated as a percentage of the total length of the small intestine from duodenum to cecum.

### Isolation of Intestinal Epithelial Cells

After overnight fasting, mice were sacrificed and the distal part of the small intestine (ileum) was removed, cut into small pieces, opened, and washed with PBS. Intestinal pieces were, then, immersed in 3 ml of 30 mM EDTA in PBS and shacked at 4°C for 45 min. Afterward, samples were spun and pellets incubated with 250 µg of collagenase (Sigma, St. Louis, MO, USA) in 5 ml HBSS at 37°C for 10 min with gentle shaking. After incubation, tissue debris was removed and cells washed and re-suspended in 1 ml Trizol for RNA extraction and stored at −80οC until used.

### Quantitative RT-PCR Analysis

qRT-PCR was carried out as previously described (35) on RNA extracted from whole spleen cells and ileum epithelial cells. After RNA extraction and purification, cDNA was synthesized using TaqMan reverse transcription reagents (Applied Biosystems, Foster City, CA, USA) according to manufacturer’s protocol. Premade Taqman primers and probes (Applied BioSystem) were used to study the expression of HPRT (Mm01545399_m1). The sequences of the primers and probes used to study expression of AMPs and associated genes are shown in Table 1 and were purchased from Metabion (Steinkirchen, Germany). Transcript levels of target genes were normalized according to the dCq method to respective mRNA levels of the housekeeping gene HPRT.

**Table 1:** List of primers.

Defa1	Forward	CCGTATCTGTCTCCTTTG
	Reverse	CGACAGCAGAGCGTGTA
	Probe	FAM-AATGGAACCTGCAGAAAGGGTCATT-BHQ1

Defa4	Forward	CCAAGAAGGGTCTGCTCT
	Reverse	GCGGGGGCAGCAGTA
	Probe	FAM-AGTTCGTGGGACTTGTGGAATACGAT-BHQ1

CRS4C	Forward	CAGGCTGTGTCTGTCTCTTT
	Reverse	TTTGGATTGCATTTGCACCTC
	Probe	FAM-CGTCATGCCCATCTTGCCCGAGA-BHQ1

CRS1C	Forward	CAGAAGGCTCTGCTCTTCA
	Reverse	GCCTTTCTTCGCACAATGG
	Probe	FAM-AAGTGCCAAGTGTGCCAGAAGTGC-BHQ1

MMP-7	Forward	CACTGAACTTCAAGAGGGT
	Reverse	CATCCTTGTCAAAGTGAGC
	Probe	FAM-AACACTCTAGGTCATGCCTTCGCA-BHQ1

Reg3γ	Forward	TGGATTGGGCTCCATGAC
	Reverse	TGAGGCTCTTGACAGGG
	Probe	FAM-ACGAATCCTTCCTCTTCCTCAGGC-BHQ1

Angiogenin 4	Forward	CTTGACACCGAAGGAC
	Reverse	TCACAACCAGACCCAGCA
	Probe	FAM-AGCCCATGTCCTTTGTTGTTGGTCT-BHQ1

### *In Vivo* Bioluminescence Imaging (BLI)

Mice pretreated with saline or paraoxon were infected by oral gavage with SL1344:*lux* (1 × 10^5^ CFUs/200 μl/mouse). At different time points, mice were imaged and bacterial dissemination was followed by *in vivo* BLI using the *in vivo* imaging system Lumina II (Caliper Life Sciences, Hopkinton, MA, USA). Data acquisition and analysis were performed using living image software (Caliper Life Sciences).

### Histological Analysis of the Ileum

Excised ileum was processed for histological analysis following established protocol (34). Tissue sections were stained with hematoxylin and eosin and images were captured with an Olympus BX51 microscope equipped with digital camera DP26 (Olympus Corporation, Tokyo, Japan). For electron microscopy study, ileum was fixed in a mixture of 2.5% glutaraldehyde and 2% formaldehyde solution (pH = 7.2 in phosphate buffer). Samples were rinsed with 0.1 M phosphate buffer, post-fixed in 1% aqueous osmium tetroxide, dehydrated in ascending series of graded ethanol, infiltrated with Agar100 epoxy, and finally embedded in the same resin where they polymerized. Blocks were trimmed and ultrathin (95–100 nm) sections were prepared on 200 mesh copper grids and contrasted with 2% uranyl acetate and lead citrate. Sections were examined and photographed with TENAI G2 Spirit Transmission Electron Microscope (FEI, Hillsboro, OR, USA). Indirect immunostaining for mucin was performed using rabbit polyclonal anti-Mucin 2(H-300 clone, Santa Cruz Biotechnology, Santa Cruz, CA, USA) followed by FITC-conjugated donkey anti-rabbit IgG (Jackson ImmunoResearch, West Grove, PA, USA), counter-stained with propidium iodide (BD Bioscience) and then examined and photographed under a Nikon C1 laser scanning confocal microscope.

### Statistical Analysis

Statistical significance between control and treated groups was analyzed by Mann–Whitney test or unpaired two-tailed Student’s *t*-test. Survival analysis was performed by Kaplan–Meier survival curves and log-rank test (Mantel–Cox). All tests were performed using GraphPad Prism software (San Diego, CA, USA). Differences between experimental groups were considered significant when *p* values were < 0.05.

## Results

### Paraoxon Exposure Reduced RBC AChE Activity and Retarded Body Weight Gain

The effectiveness of paraoxon treatment as a potent AChE inhibitor was confirmed by measuring the AChE activity in RBC. Blood from control and experimental animals was collected before the initiation of paraoxon treatment and at the end of each of the three weeks of treatment. After 1 week of paraoxon administration, AChE enzymatic activity was reduced by 50% of baseline value. This reduction was maintained during the three weeks of treatment (Figure S1A in Supplementary Material). In contrast, control group showed no significant changes in the enzyme activity during the 3 weeks of saline administration. Differences in AChE activity between the experimental and control groups were significant at all time points (*p* ≤ 0.001), confirming that paraoxon treatment resulted in AChE inhibition.

The changes in body weight over the 3-week-treatment period were also monitored. Body weights were recorded before starting the treatment with saline (control) or paraoxon and considered as a baseline. Mice were weighed weekly during the treatment and the percentage change in body weight calculated. For saline-treated animals, mean body weight gradually increased by 3.5% after the first week, 6.8% after the second week, and 7.6% after the third week of treatment (Figure S1B in Supplementary Material). In contrast, paraoxon-treated mice showed a 0.09% reduction in their weights after the first week of treatment. During the following week (week 2), mice experienced a modest increase of 1.6% of their original weights. During the third week of the treatment, their body weights remained constant compared with the week before. Taken together, the data demonstrate that animals treated with paraoxon exhibit a significant reduction in body weight gain during the period of treatment in comparison to control mice. This is thought to be due to suppressed food intake and increased hyperactivity associated with cholinergic stimulation (24, 36).

### Paraoxon Treatment Protects Against Oral, but Not Systemic, *Salmonella* Infection

**Figure 1:**
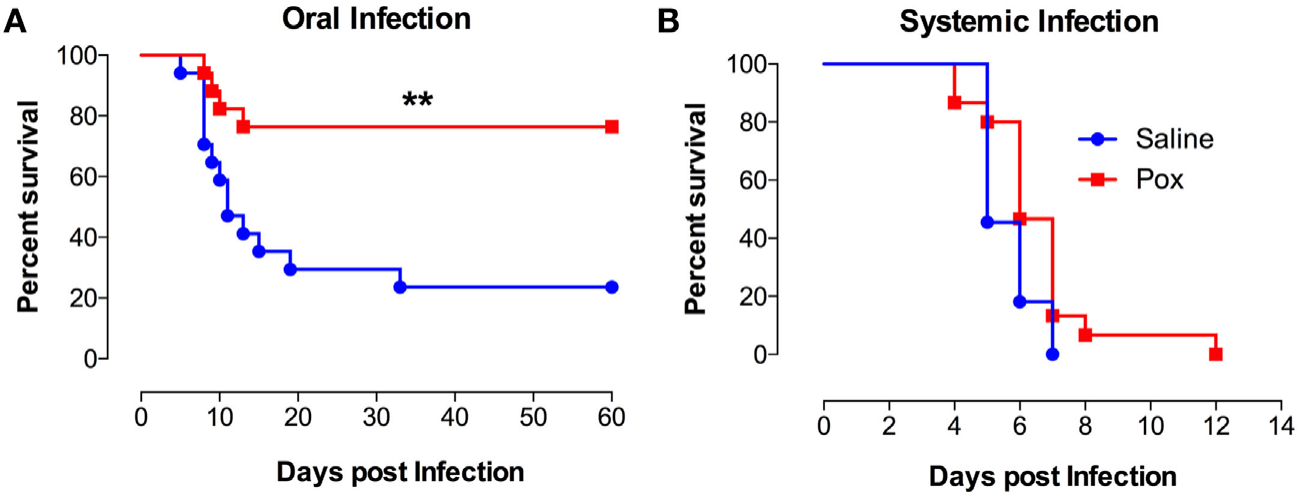
Exposure to paraoxon improves resistance to oral but not to systemic infection with virulent bacteria. Mice treated with paraoxon or saline for 3 weeks were infected with virulent *Salmonella* either **(A)** orally [1 × 10^4^ colony-forming units (CFUs)/mouse] or **(B)** i.p. (5 × 10^3^ CFUs/mouse) and followed for survival. Graphs represent the mean values ± SEM of pooled data from 2 to 3 independent experiments (*n* = 15–17/group). Asterisks denote statistically significant differences between the control and experimental groups (**p < 0.01). Log-rank (Mantel–Cox) statistical test was used for the analysis.

Following treatment with paraoxon or saline, mice were inoculated orally with SL1344, a virulent strain of *S. typhimurium* and followed for survival (Figure 1A). Mice pretreated with paraoxon survived the virulent infection, with 77% of the animals surviving up to day 60 postinfection. By contrast, only 24% of control animals survived the infection with a median survival of 11 days (Figure 1A). However, when mice were infected systemically (i.p. route), no animals of either pretreatment group could survive an infection (Figure 1B). These results suggest that activation of the cholinergic pathway potentiates immunity against a lethal infection uniquely at the intestinal mucosal interphase.

### Cholinergic Stimulation Reduces Bacterial Proliferation but Does Not Alter Mucosal Antibody Responses

**Figure 2:**
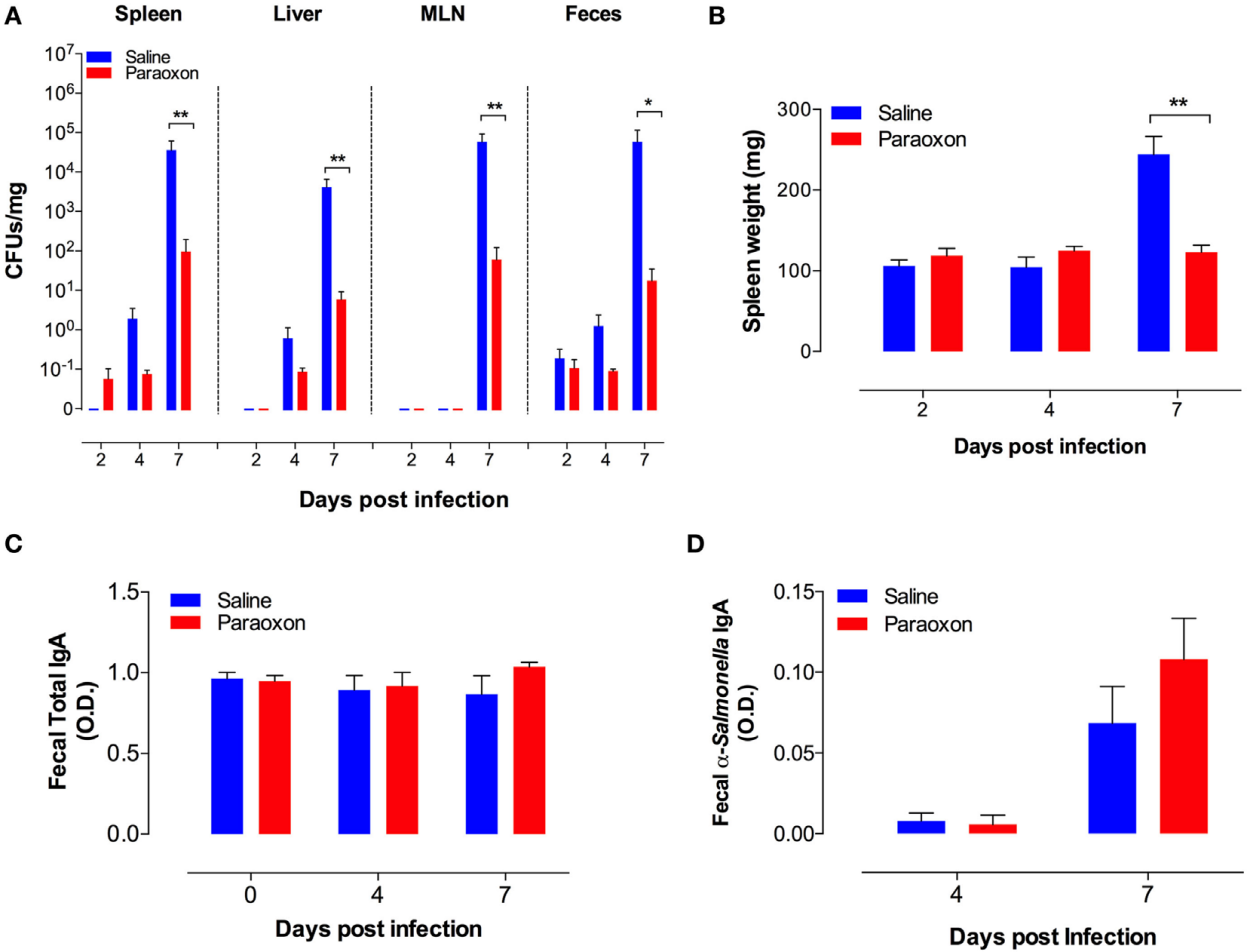
Acetylcholinesterase inhibition reduces bacterial load and prevents splenomegaly in orally infected mice without altering intestinal IgA production. Following pretreatment with saline or paraoxon, mice were infected orally with SL1344, as in Figure 1. **(A)**. Bacterial colony-forming units (CFUs) were determined in spleen, liver, mesenteric lymph nodes (MLNs), and feces at the indicated time points postinfection and expressed as CFUs/mg. **(B)** Spleen weights of infected mice. **(C)** Total fecal IgA levels and **(D)** specific fecal anti-*Salmonella* IgA levels were determined by ELISA. Graphs depict the mean values ± SEM of data from three independent experiments (*n* = 4–10/group). Asterisks denote statistically significant differences between control and experimental groups (*p < 0.05, **p < 0.01).

In order to gain further insight, we determined bacterial CFUs at mucosal sites (feces and MLNs) and systemic organs (spleen and liver) at different time points after an oral infection with *Salmonella*. As shown in Figure 2A, oral *Salmonella* infection of control mice resulted in time-dependent increase in bacterial CFUs in all the tissues tested. Over a period of only 7 days, the extent of bacterial proliferation increased by a factor of at least 10^4^-fold in saline-pretreated mice. The bacterial load in the systemic organs (spleen and liver) reached 10^4^–10^5^ CFUs/mg, which is equivalent to the level (>10^7^ CFUs/organ) that invariably leads to septic shock in *Salmonella*-infected mice (32, 37). By contrast, paraoxon-pretreated mice demonstrated an increased resistance to *Salmonella* growth in all tissues, with mean bacterial loads being 100- to 1,000-fold lower than in control mice. Moreover, while control mice developed infection-associated splenomegaly by day 7, paraoxon-pretreated mice did not (Figure 2B). We wondered if the resistance to infection exhibited by paraoxon-treated animals was associated with enhanced mucosal antibody response to *Salmonella*. Fecal pellets were collected immediately after treatment (saline or paraoxon) and at day 4 and 7 postinfection with the virulent *Salmonella* strain SL1344. Paraoxon treatment had no effect on total IgA levels (Figure 2C). Moreover, there was no significant change in total IgA levels during the course of the infection in any group. We also determined the level of *Salmonella*-specific IgA at 4 and 7 days postinfection (Figure 2D). Anti-*Salmonella* IgA levels were increased at day 7 postinfection in both experimental groups. Although paraoxon-pretreated mice exhibited slightly higher levels of *Salmonella-*specific IgA than saline group, the differences were not statistically significant (Figure 2D). Finally, in order to firmly assess whether alterations in bacterial shedding in the feces of paraoxon-pretreated mice could account for the observed differences, we determined in a separate series of experiments the fecal CFUs at days 1, 2, and 3 postinfection. The mean ± SEM CFUs/mg feces for saline vs. paraoxon groups were 2.3 ± 0.6 vs. 1.5 ± 0.6, 2.6 ± 0.7 vs. 1.3 ± 0.4, and 2.0 ± 1.1 vs. 3.5 ± 3.2, at days 1, 2, and 3 postinfection, respectively. These results confirmed that there were no significant changes in bacterial shedding between the two experimental groups at these early time points following oral infection. These findings suggest that protection from lethal infection is correlated with an efficient control of bacterial growth at the mucosal interphase and inhibition of bacterial dissemination to systemic organs.

### Paraoxon-Pretreated Mice Exhibit Reduced Level of Activation of Systemic Immune Responses Following *Salmonella* Infection

**Figure 3:**
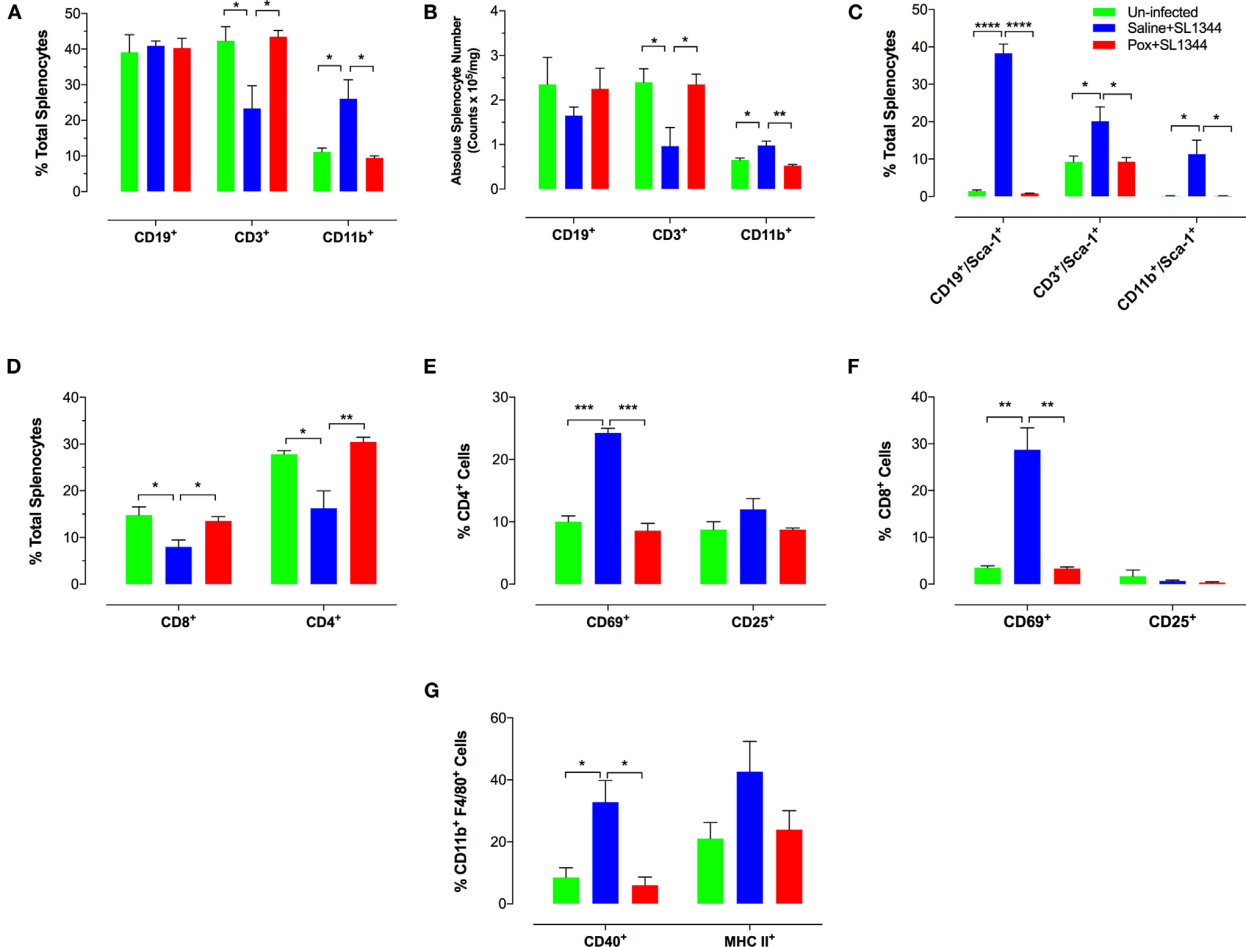
Immunophenotypic analysis of spleen cell subpopulations following oral infection with virulent *Salmonella*. Saline or paraoxon-pretreated mice were orally infected with SL1344 and analyzed by FACS 7 days later. **(A,B,D)** Spleen cells were immunophenotyped using lineage specific markers for B cells (CD19), myeloid cells (CD11b), T cells (CD3), and T cell subsets (CD4, CD8). **(C)** Relative expression of Sca-1 on the three major subpopulations was uses to assess general activation status. **(E,F)** Expression levels of CD69 and CD25 on CD4 and CD8 T cell subsets. **(G)** Expression of CD40 and MHC class II proteins on splenic CD11b^+^F4/80^+^ macrophages was also analyzed. Data are mean values ± SEM from a representative of three independent experiments (*n* = 4–5/group). Asterisks denote statistically significant differences between groups (*p < 0.05, **p ≤ 0.01, ***p < 0.001, ****p < 0.0001).

We next assessed the alterations in spleen cell composition following *Salmonella* infection in control and paraoxon-pretreated mice. At day 7 post oral infection with SL1344, there was a significant increase in the percentage of splenic myeloid cells in saline-pretreated infected mice compared to non-infected animals (Figure 3A) (mean% CD11b^+^ cells: 26.1 ± 5.3 vs. 11.2 + 1.1; *p* = 0.033). This is also observed in terms of absolute cell counts in the spleen (Figure 3B). The increase in myeloid cells was mirrored by a corresponding decrease in the percentage of splenic T lymphocytes in infected animals (mean = 42.3 ± 3.9 vs. 23.3 ± 6.4; *p* = 0.043). No changes were observed in the splenic B cell population following infection (Figure 3A). The ratios of spleen cell subpopulations in infected paraoxon-treated mice were very similar to non-infected animals, reflecting the fact that systemic spread of the bacteria has been controlled. We also analyzed the level of expression of different activation markers on the various spleen cell populations. As shown previously, Sca-1 (Ly6A/E) expression is upregulated on lymphoid and myeloid cells by IFNα/β and IFNγ (33, 38). Spleen cells from non-infected mice expressed very low levels of Sca-1 protein (Figure 3C). However, Sca-1 expression was clearly increased in B and T lymphocytes as well as myeloid cells in the spleen of infected mice (Figure 3C). Interestingly, splenic cell populations from infected paraoxon-pretreated mice showed no significant changes from those of non-infected mice in their Sca-1 expression (Figure 3C).

Analysis of splenic T cell populations and their activation status confirmed the above pattern. Thus, the percentage of CD4 and CD8 T cells was similar in non-infected and paraoxon-pretreated infected mice and this differed markedly from that of saline-pretreated infected animals (Figures 3D,F). In line with the data in Figure 3A, the decreased percentage in splenic T cells was reflected by a decrease in both the CD4 and CD8 subpopulations (Figure 3D). Moreover, the percentage of cells expressing CD69, a marker of recently activated T cells (39, 40), was increased in both CD4 and CD8 T cells of saline-pretreated infected mice, but not the other two experimental groups (Figures 3E,F). Finally, analysis of activation marker expression on CD11b^+^F4/80^+^ splenic macrophages revealed evidence of upregulated CD40 and MHC class II protein expression on cells from saline-pretreated infected mice only but not on non-infected or paraoxon-pretreated infected experimental groups (Figure 3G). Taken together, the results of the phenotypic analysis confirm that stimulation of the cholinergic pathway protects against an oral infection with *Salmonella* and prevents its dissemination to systemic targets.

### Low Levels of Splenic Inflammatory Cytokines in Paraoxon-Treated Mice Following Infection

**Figure 4:**
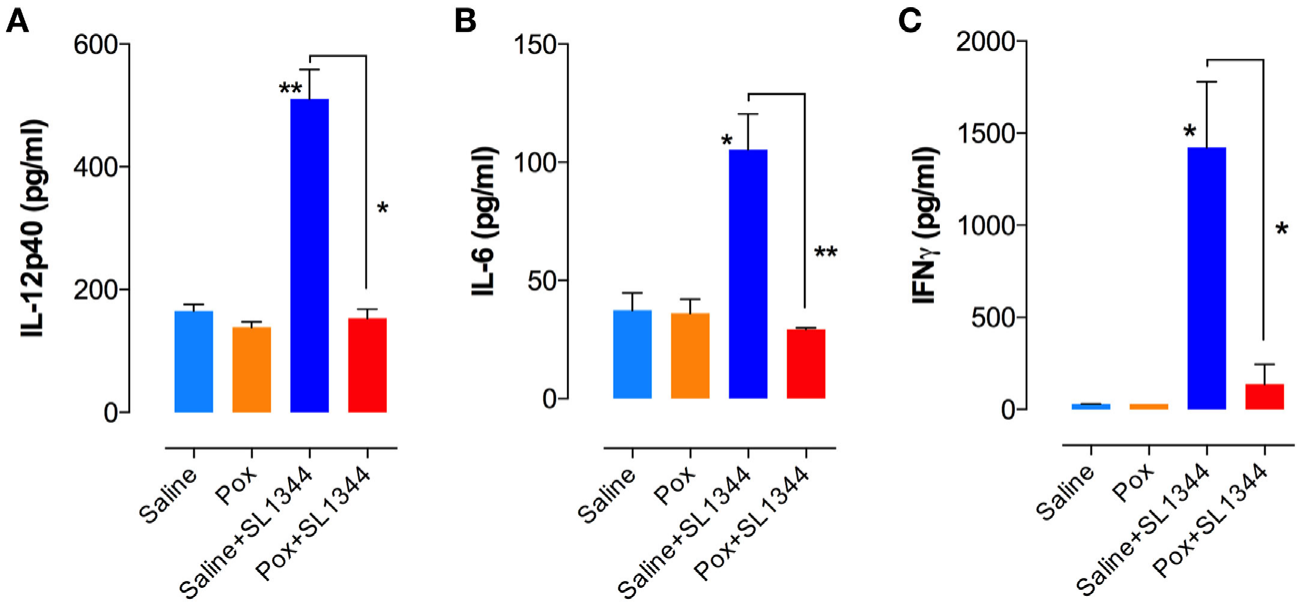
Cytokine production in *Salmonella*-infected mice. Spleen cells prepared as in Figure 3 were cultured overnight in the absence of additional stimuli. Cell-free culture supernatants were collected and tested for the presence of **(A)** IL-12p40, **(B)** IL-6, and **(C)** IFNγ by specific ELISAs. Graphs depict the mean values ± SEM from a representative of two independent experiments (*n* = 4–5/group). Asterisks denote statistically significant differences between control and experimental groups (*p < 0.05, **p ≤ 0.01).

We next assessed cytokine production by *ex vivo* whole spleen cells from non-infected and infected mice. At day 7 postinfection, splenocytes from saline or paraoxon-pretreated groups were cultured *ex vivo* without any further stimulation and cytokine content was examined in 24 or 48-h culture supernatants. Significant production of IL-12/IL-23p40, IL-6, and IFNγ was observed only in saline-pretreated, *Salmonella*-infected mice (Figures 4A–C). Animals pretreated with paraoxon and then infected orally with virulent *Salmonella* survived the infection but had no evidence of pro-inflammatory cytokine production in the spleen. Indeed, the levels of cytokines in this group of animals were very similar to the background levels detected in non-infected mice. Thus, paraoxon-induced protection against oral *Salmonella* infection does not appear to be due to a more effective systemic immune responsiveness.

### Paraoxon Pretreatment Induced Phagocytic Activity of Splenic Myeloid Cells but Does Not Increase Intestinal Transit

**Figure 5:**
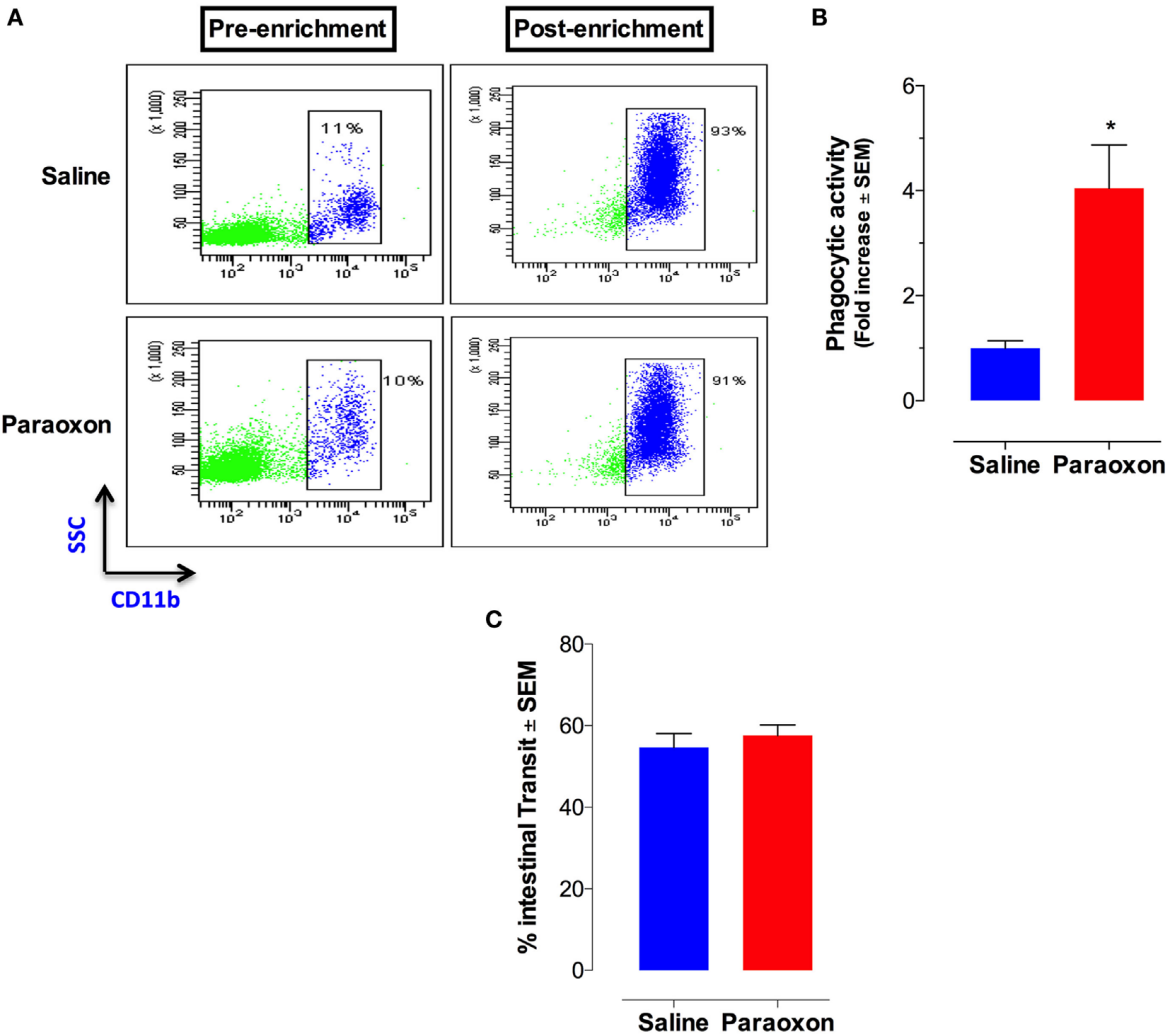
Acetylcholinesterase inhibition increases phagocytic activity of splenic macrophages but does not alter intestinal motility. Spleen cells were prepared as described above and used to isolate CD11b^+^ cells by enrichment on magnetic columns. **(A)** FACS analysis of splenic CD11b^+^ cells before and after enrichment. **(B)** Purified CD11b^+^ cells were assayed for phagocytic activity. The graph depicts the fold increase in phagocytic activity of purified macrophages from paraoxon-pretreated mice as compared to saline control. Asterisks denote statistically significant differences between groups (*p < 0.05). **(C)** In another set of experiments, intestinal motility was assessed 72 h after the last dose of paraoxon/saline, as described in Section “Materials and Methods.” Intestinal transit was measured and calculated as percentage of the total length of the small intestine in each animal. Graphs depict mean values ± SEM of three mice/group from a representative one of two independent experiments.

Given the central role of macrophages in *Salmonella* infections (41), we assessed whether paraoxon pretreatment had any effect on phagocytic cell activity. Splenic myeloid (CD11b^+^) cells were purified at the end of the 3-week paraoxon, or saline, treatment by positive selection on magnetic columns. The purity of CD11b^+^ cells was verified by flow cytometry to be >90% (Figure 5A). Purified myeloid cells were then assessed for their capacity to phagocytose fluorescein-labeled *E. coli* BioParticles. The findings showed a 3.5-fold enhancement in the phagocytic activity of myeloid cells from paraoxon-pretreated mice compared to the control group (Figure 5B). These results suggest that stimulation of the cholinergic pathway could potentially improve the readiness of myeloid cells to phagocytose bacteria.

Innervation of the GI tract by the vagus nerve is known to enhance motility (42, 43). We reasoned that the enhanced survival following a lethal oral infection in paraoxon-treated mice could be attributed to enhanced intestinal motility that would lead to a faster bacterial clearance from the intestine. To test this possibility, mice were pretreated with saline or paraoxon for 3 weeks, as per protocol, followed by Evan’s blue dye administration. Mice were sacrificed 15 min later and the distance traveled by the dye in the small intestine was measured. The results of this analysis showed no difference in the intestinal motility between the control and paraoxon groups (Figure 5C). This indicates that the enhanced survival in paraoxon-treated animals cannot be attributed to a faster intestinal elimination of bacteria.

### Failure of Paraoxon Treatment to Protect Against a Systemic Infection Is Linked to a Failure to Control Bacterial Proliferation

Next, we wanted to assess whether paraoxon exposure can modulate the immune response to systemic infection. Following treatment with saline or paraoxon, mice were infected intraperitoneally (i.p.) with virulent *S. typhimurium* (SL1344) and examined for immune responsiveness at 2 and 20 h later. The bacterial loads in the peritoneal cavity and target organs like spleen and liver were similar in animals regardless of the pretreatment with saline or paraoxon (Figures S2A–C in Supplementary Material). Moreover, the fact that bacterial CFUs in the spleen and liver rose by more than 2 logs over a 20-h period indicates profoundly inadequate immune responses. The extent of splenomegaly was also recorded at these early time points, but the data indicated no differences between saline and paraoxon-treated groups (Figure S2D in Supplementary Material). To gain further insight into the innate immune response to infection, the levels of IL-12p40 were determined in serum as well as in supernatants of *ex vivo*-cultured splenocytes (Figures S2E,F in Supplementary Material). The IL-12p40 levels peaked after 2 h of infection in both saline and paraoxon-treated mice and were ~10-fold higher in serum than in the supernatants collected from cultured splenocytes. Although IL-12p40 levels were slightly higher in paraoxon-treated group, this difference did not reach statistical significance (Figures S2E,F in Supplementary Material).

### Cholinergic Stimulation Does Not Modulate the Immune Response to Systemic Infection With an Attenuated Strain of *Salmonella*

Given that *S. typhimurium* SL1344 is a highly virulent strain with LD_50_ of <10 CFUs when given systemically (44), we reasoned that the high degree of virulence may mask any beneficial effect that the activation of the cholinergic pathway could have on the antimicrobial immune response. Therefore, we examined if paraoxon treatment could improve the immune response to a non-virulent strain of *Salmonella* when given systemically. Animals pretreated with saline or paraoxon were infected i.p. with the attenuated *Salmonella* strain BRD509E. Bacterial loads in spleen, liver, and peritoneal cavity were determined 2 and 7 days later (Figure S3A–C in Supplementary Material). At the early time point, a small (~2-fold) but significant reduction in spleen (means ± SEM of 185 ± 40 and 50 ± 26 CFUs/mg for saline and paraoxon groups, respectively) and liver (111 ± 20 and 42 ± 13 CFUs/mg, respectively) bacterial loads was noted in paraoxon-pretreated animals compared to controls (Figures S3A,B in Supplementary Material). This was paralleled by a significantly lower degree of splenomegaly (Figures S3D in Supplementary Material). By day 7 postinfection, however, all of the measured parameters were similar in both experimental animal groups.

To further investigate the effect of paraoxon treatment on immune response to systemic infection with BRD509, we analyzed changes in cellularity and activation status of spleen lymphocytes by flow cytometry. This analysis, which was carried out at 2 as well as 7 days postinfection, revealed no gross alterations in spleen B and T cell composition between saline and paraoxon-pretreated mice (Figures S4A,C in Supplementary Material). Moreover, lymphocyte activation status was assessed by determining the level of Sca-1 expression. While B cells exhibited a transient reduction in the level of Sca-1 expression at day 2, no significant changes in activation status was observed at day 7 postinfection (Figure S4D in Supplementary Material) or indeed in the T cell compartment at any time (Figure S4B in Supplementary Material). The transient reduction in B cell activation observed at day 2 postinfection is most likely due to the transient and small decrease in bacterial CFUs in target organs that is observed at that time (Figures S3A,B in Supplementary Material). The extent of macrophage recruitment into the peritoneal cavity at 2 and 7 days post i.p. infection was also assessed. The findings showed no difference in the peritoneal macrophage response to infection between saline and paraoxon-pretreated animals (data not shown).

In order to assess responsiveness to infection, we also analyzed the production of inflammatory mediators by splenocytes of *Salmonella*-infected mice. After overnight culture of splenocytes, cell-free supernatants were tested for NO, IL-12p40, IL-6, and IFNγ content (Figure S5 in Supplementary Material). No significant differences in the production of any of infection-induced mediators were found between paraoxon- and saline-pretreated groups at either time point. Despite the fact that IFNγ levels were detected at somewhat higher levels in paraoxon-pretreated mice (Figure S5D in Supplementary Material), the differences did not reach statistical significance. Overall, we conclude that pretreatment with paraoxon did not afford any protection to systemically administered *Salmonella*, regardless of the degree of virulence of the bacterial strain.

### AChE Inhibition Delays Systemic Dissemination of Oral *Salmonella*

**Figure 6:**
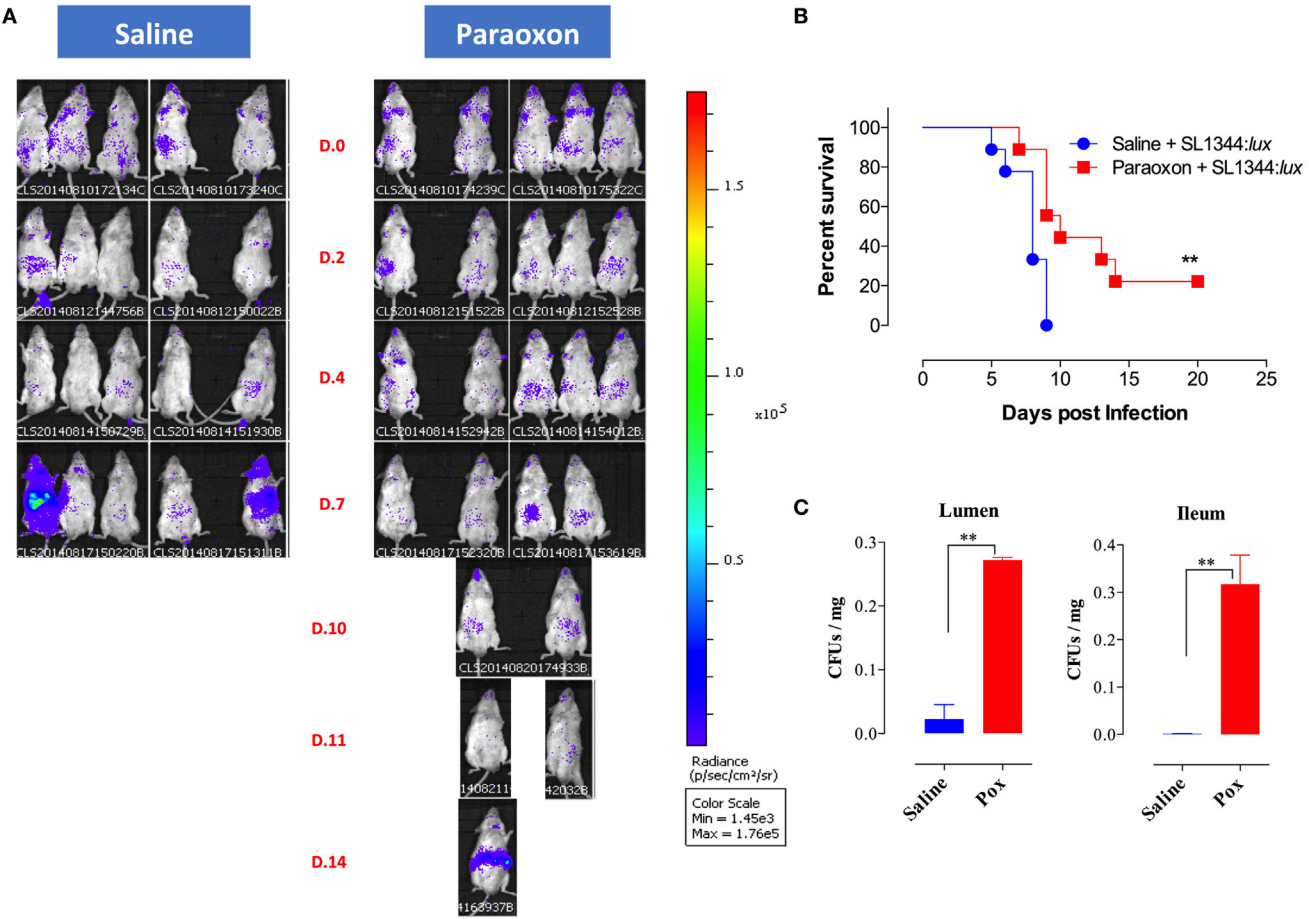
*In vivo* bioluminescent imaging of *Salmonella*-infected mice. Following saline or paraoxon pretreatment, mice were orally infected with SL1344:*lux* [1 × 10^5^ colony-forming units (CFUs)/mouse]. **(A)** Mice were imaged ventrally starting at 3 h postinfection (Day 0 or D.0) and followed until day 20 postinfection. Data are representative of two independent experiments (five mice/group). Color scale ranges from 1,450 (purple) to 176,000 photons/sec/cm^3^/sr (red). **(B)** Animals were also followed for survival. The graph represents the mean values ± SEM of pooled data from two independent experiments (*n* = 9 mice/group). Asterisks denote statistically significant differences [log-rank (Mantel–Cox) statistical test] between the control and experimental groups (**p < 0.01). **(C)** Bacterial loads in the small intestine following oral infection with SL1344. Bacterial CFUs were determined in the lumen of the small intestine and in the ileum at days 1 and 3 postinfection, respectively. Graphs depict mean values ± SEM of 3–4 mice/group from a representative one of two independent experiments. Asterisks denote statistically significant differences between the indicated groups (**p < 0.01).

Given the lack of protection to systemic infection, we focused our investigation on how paraoxon pretreatment protected against oral *Salmonella*. After 3 weeks of treatment with saline or paraoxon, mice were orally inoculated with a relatively high dose (1 × 10^5^ CFUs/mouse) of bioluminescent strain of SL1344 (designated SL1344:*lux*) and bacterial dissemination was followed by live imaging. Mice were imaged at different time points until they succumbed to the infection, between day 0 and day 20 postinfection (Figure 6). Initially, all mice in both groups showed a mostly localized distribution of the bacteria in the intestinal region (Figure 6A). Starting at day 4–5 postinfection, some animals from the control group exhibited dissemination of bacteria to systemic organs and began to succumb to the infection by day 5 (Figure 6B). By day 9, all animals in the saline group died due to disseminated *Salmonella* infection. In contrast, paraoxon-treated mice showed a delayed dissemination of bacteria to systemic organs that was not apparent until day 7–8 postinfection. This led to a significantly enhanced survival in this group of animals (22% of mice survived up to day 20) despite the higher dose of SL1344 used in these studies. In a separate set of experiments, we determined the bacterial load in the lumen of the small intestine 24 h postinfection as well as in the ileum region at day 3 postinfection. The data showed significantly higher *Salmonella* bacterial loads at both sites in paraoxon-treated mice (Figure 6C). These data suggest that bacterial translocation across the intestinal barrier is inhibited or delayed in paraoxon-treated mice, which could account for their enhanced survival.

### Expression of AMPs in the Intestinal Epithelium

**Figure 7:**
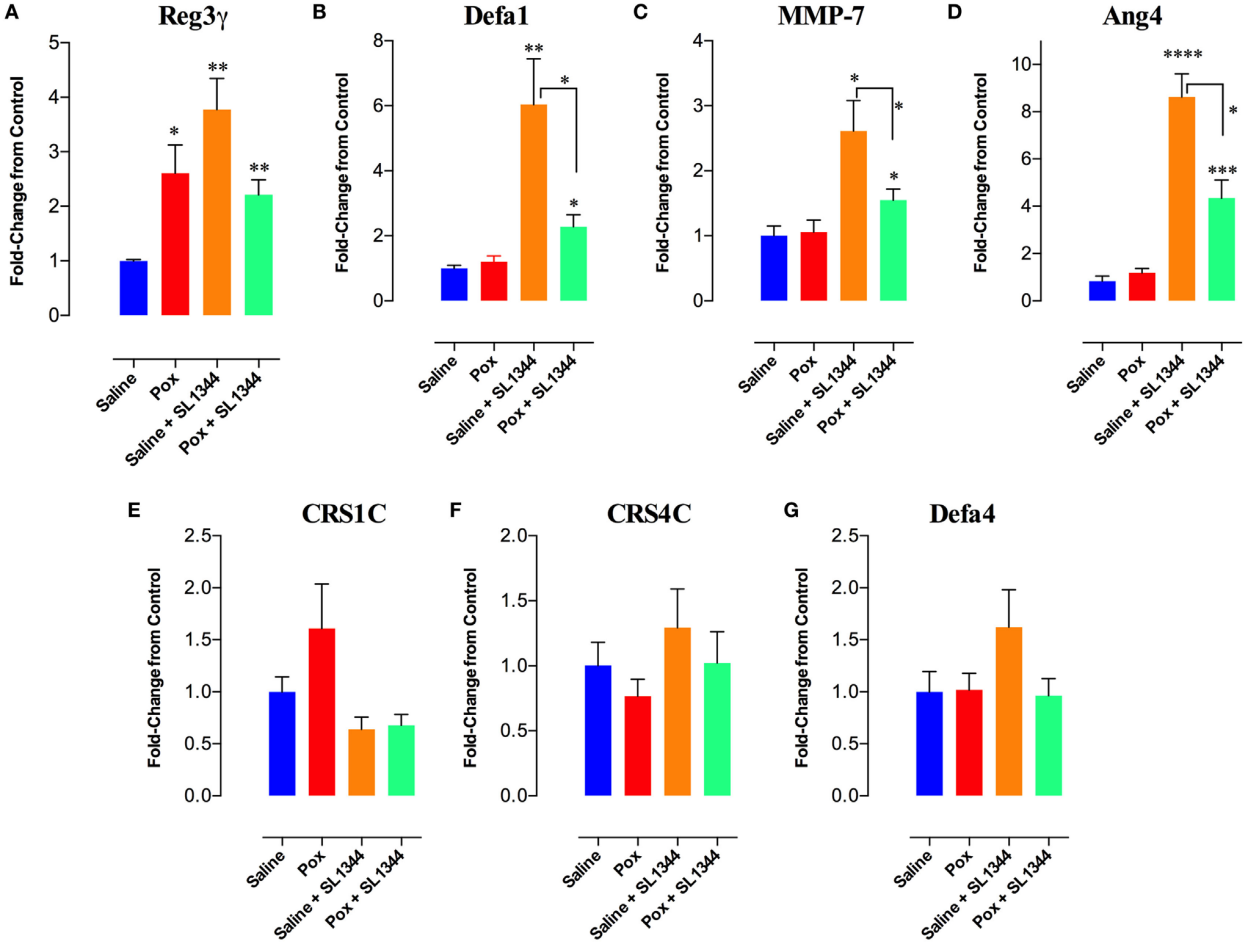
Analysis of gene expression of antimicrobial peptides (AMPs) in intestinal epithelial cells. The last part of the small intestine (ileum) was aseptically removed from mice pretreated with paraoxon or saline. The ileum was also obtained from mice pretreated with saline or paraoxon, and orally infected with virulent *Salmonella* [1 × 10^4^ colony-forming units (CFUs)/mouse] at day 4 postinfection. RNAs were extracted, converted to cDNA, and ran for qRT-PCR. **(A–G)** Graphs depict the fold change in the expression of the different AMPs in comparison to control saline group. Graphs depict mean values ± SEM of cumulative data from two independent experiments (*n* = 6/group). Asterisks denote statistically significant differences between groups (*p < 0.05, **p ≤ 0.01, ****p < 0.0001).

The intestinal epithelium is the first barrier that bacteria encounter following an oral infection. Thus, we investigated whether paraoxon treatment has any effect on the intestinal mucosa. First, we studied the expression of AMPs in the intestinal epithelium (ileum) of uninfected saline or paraoxon-pretreated mice. Our results showed that paraoxon treatment did not significantly alter the expression of Defa1 (defensin α1), Defa4 (defensin α4), CRS1C (cryptdin-related sequence 1 C), CRS4C (cryptdin-related sequence 4 C), Ang-4, or the enzyme MMP-7 (matrix metalloproteinase-7) in ileal epithelial cells (Figures 7B–G). Surprisingly, however, the expression of RegIII-γ (Regenerating islet-derived protein 3 gamma) was 2.6-fold higher in paraoxon-treated mice than in control animals indicating that AChE inhibition induces specifically the expression of RegIIIγ (Figure 7A).

Next, the expression of these genes was analyzed after 4 days of infection with SL1344. In saline-pretreated mice, SL1344 infection resulted in a significant induction of Reg3γ (3.8-fold), Defa1 (6-fold), MMP-7 (2.6-fold), and Ang-4 (8.6-fold) expression (Figures 7A–D). Infection of paraoxon-pretreated mice also led to a significant enhancement in the expression of Defa1 (2.3-fold), MMP-7 (1.6-fold), and Ang-4 (4.3-fold) antimicrobial proteins, but not Reg3γ which was already upregulated by paraoxon treatment. Intriguingly, *Salmonella* infection of paraoxon-pretreated mice appeared to attenuate the extent of gene expression of these antimicrobial proteins compared to the infected saline-pretreated group (Figures 7A–D).

### Morphological Analysis of the Intestinal Mucosa Revealed an Induction of Mucus Secretion Following AChE Inhibition

**Figure 8:**
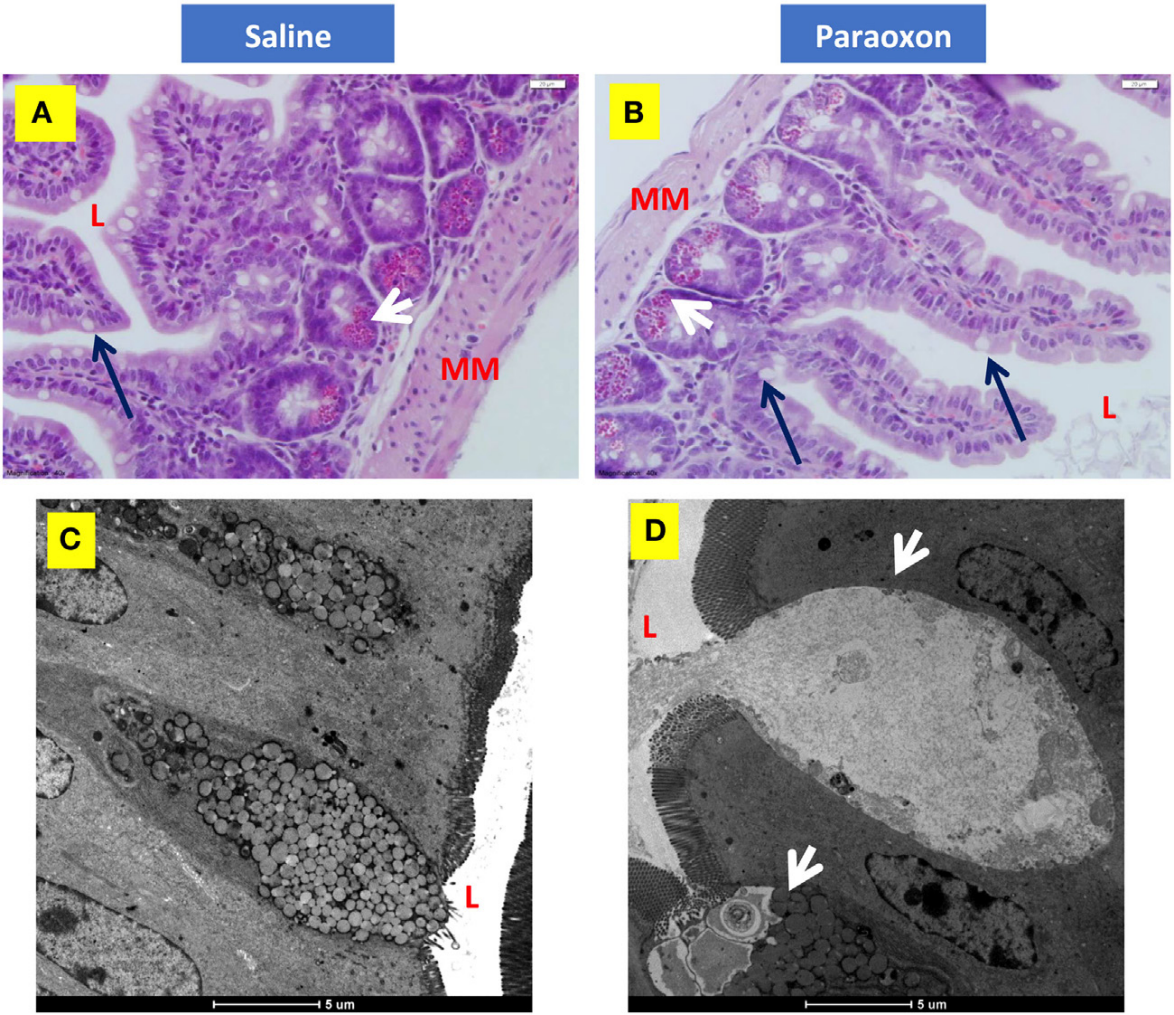
Paroxon treatment induces intestinal goblet cells (GC) degranulation. Ileum sections from **(A)** saline- or **(B)** paraoxon-pretreated mice were stained with hematoxylin and eosin. GC (black arrows) can be seen between the columnar absorbing cells. The cytoplasm of Paneth cells (white arrows) at the bottom of the intestinal crypts is full of acidophylic granules. Images were taken at 400× magnification. L, intestinal lumen; MM, muscularis mucosa. **(C,D)** Transmission electron micrographs of ileal epithelium from saline- or paraoxon-pretreated mice. **(C)** GC (arrowhead) in saline-treated animals presented with a distended apical part that contains large rounded mucous globules of moderate electron density. **(D)** In contrast, GC from paraoxon-treated mice showed either an active degranulation of their granules to the intestinal lumen (L) or completely degranulated cells. Original magnification of the micrographs 4,200×.

Finally, we investigated whether paraoxon treatment induces any morphological changes at the level of the intestinal mucosa that could affect the translocation of bacteria from the intestinal lumen to the submucosa. Light microscopy examination of the ileum revealed an intestinal mucosa with villi and crypts (Figures 8A,B). Tissue sections from paraoxon-treated mice revealed no obvious morphological changes. However, in some areas we could observe a reduced number of granules in the cytoplasm of PC located in the crypts and GC seemed to have their apical part opened to the lumen (Figure 8B; arrows).

Ultrastructural examination of the intestinal epithelium of saline-treated mice showed the presence of columnar epithelial cells with numerous apical microvilli and GC. GC presented with an apical part distended with large rounded mucous globules (Figure 8C). However, in paraoxon-treated group, intestinal GC appeared in the process of degranulation or almost depleted of the mucous globules that were released to the lumen of the intestine (Figure 8D; arrowheads). This release appears to be associated with the disruption of the apical cytoplasmic membrane and release of cytoplasmic fragments.

**Figure 9:**
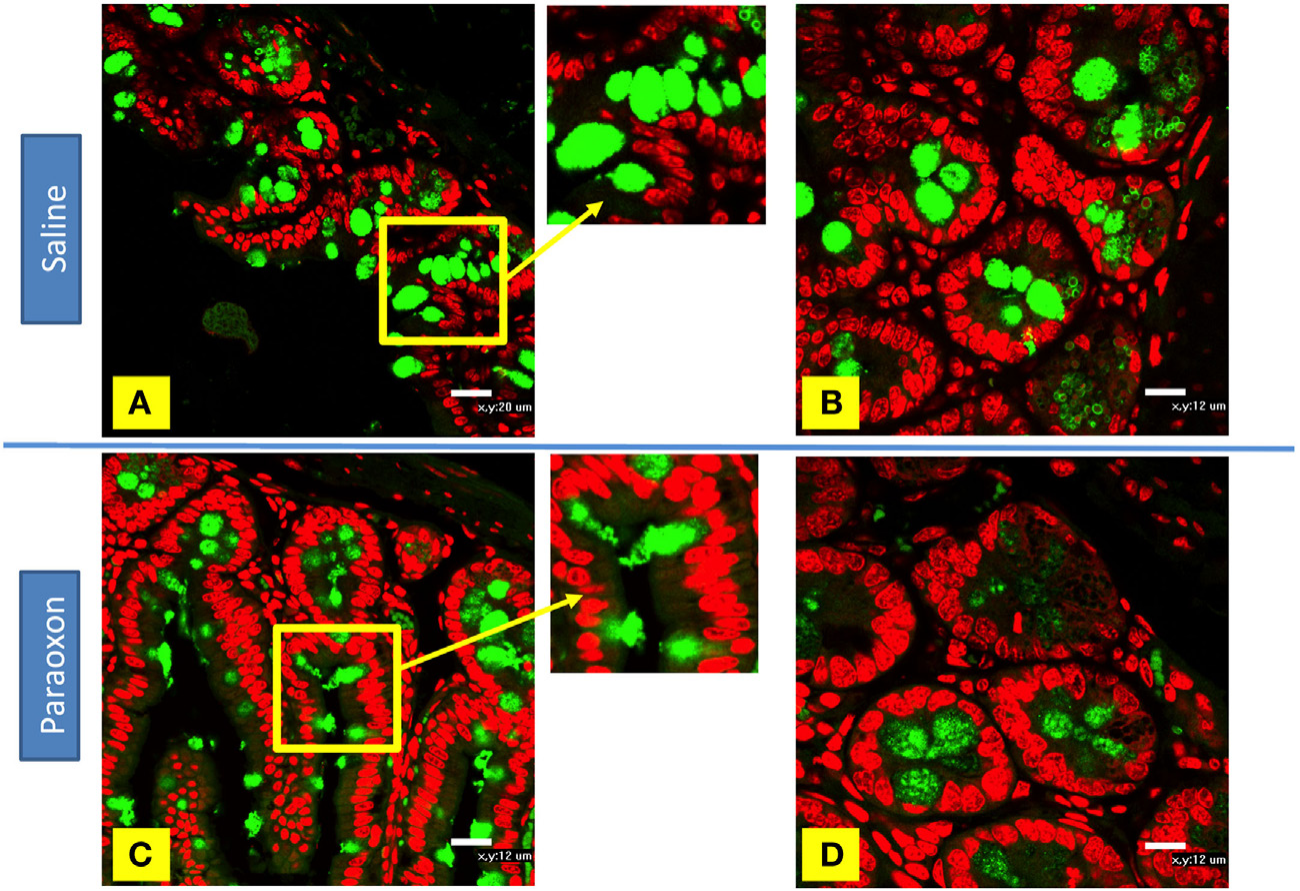
Examination of mucin staining in goblet cells (GC) by confocal microscopy. **(A–D)** Light confocal micrographs of ileum showing immunofluorescence of mucin-containing GC (green) in saline **(A,B)** and paraoxon-treated mice **(C,D)**. Figure bars indicate 20 µm **(C)** or 12 mm **(D)**. All photos are representative of two individual experiments with four mice/group/experiment.

Goblet cells degranulation was also analyzed immunohistochemically using an anti-mucin antibody (Figures 9A–D). The distal region of the small intestine (ileum) in saline-treated mice showed rounded positive cells with dense staining in their cytoplasm along the intestinal epithelium (Figures 9A,B). However, equivalent tissue sections from paraoxon-treated animals revealed GC with less dense staining and with their apical region being open to the intestinal lumen to where their mucus is released (Figures 9C,D). Therefore, mucin staining confirms that paraoxon treatment appears to induce the degranulation of GC.

**Figure 10:**
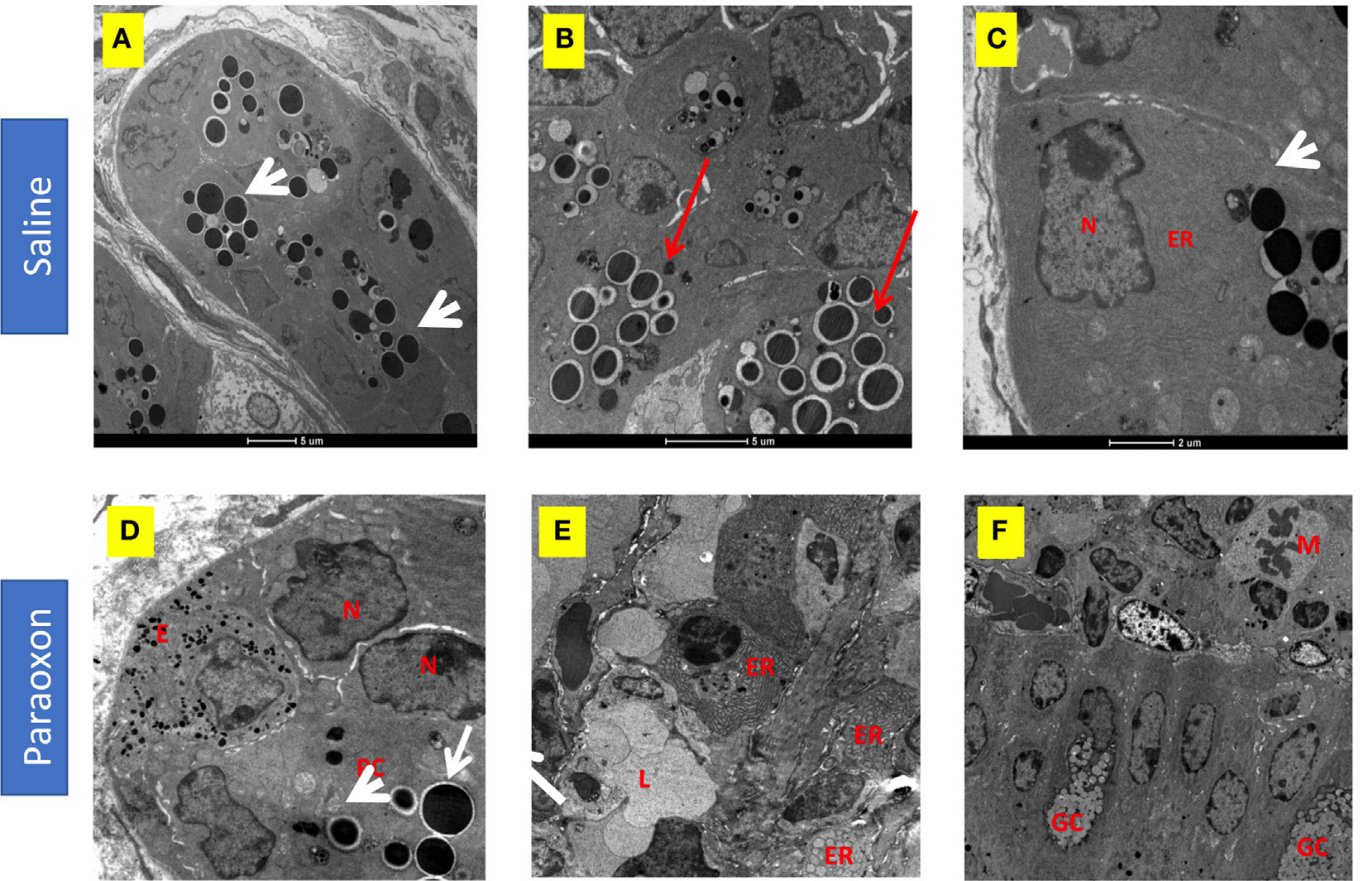
Effect of paraoxon treatment on Paneth cells (PC). Electron micrograph of PC in the crypt base of the small intestine of **(A–C)** saline or **(D–F)** paraoxon-pretreated mice. In control saline group, **(A)** the cytoplasm of PC is filled with large spherical granules (arrowhead) **(B)** some of them surrounded by clear halos (red arrows). **(C)** Higher magnification shows a PC with abundant endoplasmic reticulum (ER) in the proximity of the nucleus and the presence of secretory granules (arrowhead). **(D)** In paraoxon-treated mice, PC contain less electron-dense spherical granules of different sizes (white arrow). The PC is next to an enteroendocrine cell **(E)** containing small electron-dense granules. A group of cells in the intestinal crypts with enlarged ER and lack of granules is seen **(E)**. In panel **(F)**, mitotic cells (M) can be observed approaching the intestinal epithelium; (L) Intestinal lumen, (N) nucleus, (GC) goblet cell. Micrographs original magnification: **(A)** 2,900×, 4,200× **(B)** 9,300× **(C)**, 8,200× **(D)**, 4,800× **(E)**, and 2,900× **(F)**.

Paneth cells were seen in groups at the bottom of the crypts of Lieberkuhn in the intestine. In control mice, these cells were filled with large spherical, electron dense, granules that in some cases were surrounded by clear halos (Figures 10A–C). The basal part of these PC contained abundant endoplasmic reticulum (ER), mitochondria, and a nucleus with a prominent nucleolus (Figure 10C). In the paraoxon group, PC were found less abundant and with fewer electron-dense granules of various sizes compared to the saline group (Figure 10D). Additionally, numerous cells with enlarged ER but with no or small granules were seen (Figure 10E). Moreover, abundant mitotic figures were observed in this experimental group, which could indicate the differentiation of new PC or GC (Figure 10F).

## Discussion

Acetylcholinesterase is an enzyme responsible for terminating the action of the neurotransmitter acetylcholine. Inhibition of this enzyme leads to an increase or accumulation of ACh in the synaptic clefts, inducing a cholinergic stimulation that is widely used for the treatment of neurodegenerative diseases such as Alzheimer’s and myasthenia gravis (45, 46). Additionally, cholinergic stimulation by AChE inhibitors can modulate the immune response to autoantigens in different animal models of diabetes (24, 47). In this study, we used paraoxon, a highly specific AChE inhibitor, to regulate the immune response to an infection caused by *Salmonella enterica* serovar Typhimurium (*S. typhimurium*). Previously, we demonstrated that exposure to paraoxon protects against an oral infection by a virulent strain of *Salmonella*, SL1344 (25). The current study is aimed at characterizing the mechanisms by which the cholinergic pathway affords resistance to a lethal bacterial infection. In contrast to the oral route, we demonstrate that cholinergic stimulation does not afford any protection to intraperitoneal route infections either by virulent or attenuated strains of *Salmonella*. Moreover, the observed protection against oral infections is not linked to an enhancement of systemic, adaptive immune responses. Instead, resistance to infection appears to be mediated by improved innate immune responses at the level of intestinal mucosa. Specifically, we demonstrate that cholinergic stimulation induces the degranulation of secretory cells, including PC and GC, in the small intestine, which acts collectively to improve antimicrobial defenses at the intestinal barrier, thereby preventing the entry and dissemination of oral pathogens to systemic organs.

Evaluation of the effect of cholinergic stimulation on the immune response to a systemic infection with the virulent, wild-type, strain of *S. typhimurium* (SL1344) showed that the pretreatment did not result in a reduction of the bacterial burden in the peritoneal cavity or target organs of infected animals and, consequently, did not improve host survival. We also tested a systemic infection using the attenuated *aroA^−^*/*aroD^−^* mutant *Salmonella* strain, BRD509E. This is a well-established model where bacterial growth continues for a limited number of multiplication rounds for 7–10 days after which the host is able to clear the bacteria, usually by 3–4 weeks, thereby establishing sterile immunity (44). Using this model, our findings showed that stimulation of the cholinergic pathway results in about 50% reduction in the bacterial burden in systemic organs. This was observed only at an early time point (day 2), as no significant differences were seen at day 7 postinfection. It is likely that the lower bacterial burden in the spleen at day 2 postinfection could be due to the observed enhanced phagocytic activity of splenic macrophages induced by the cholinergic pathway. Previously, another study reported enhanced phagocytic potential observed in macrophages following ACh and vagus nerve stimulation (48). Therefore, it is possible that bacteria arriving to the spleen of paraoxon-pretreated mice could initially encounter more phagocytic macrophages that would limit, at least transiently, their proliferation.

Studies on the effect of the cholinergic anti-inflammatory pathway in live bacterial infection models are limited. The production of pro-inflammatory cytokines and neutrophil recruitment to the site of the infection are vital to minimize pathogen proliferation, and facilitate its clearance, as well as to protect the host. Inhibition of the inflammatory response causes a retarded immune response and results in increased bacterial load in the organs, failure to clear the bacteria and ultimately worsen the host survival. An early study that used a septic peritonitis model, induced by intraperitoneal inoculation of live *E. coli*, showed that stimulation of the cholinergic anti-inflammatory pathway by nicotine administration resulted in impairment of bacterial clearance and survival (19). Moreover, when α7 nAChRs*^−^*^/^*^−^* mice were used in this model, nicotine effect was reversed and bacterial load was reduced (49). A more recent study using a systemic infection with *Francisella turalrensis* reported that activation of the cholinergic anti-inflammatory pathway failed to improve animal survival (50). These results are in agreement with our present data showing that, unlike the case with oral infections, cholinergic stimulation did not improve the immune response to a systemic *Salmonella* infection. Therefore, we can conclude that the inhibition of AChE increases resistance to oral but not systemic bacterial infections.

It is well known that ACh stimulates gut functions like acid production and intestinal motility (51). Several studies have shown *in vitro* (52) and *in vivo* (42, 43) that AChE inhibitors induce an increase in gastrointestinal motility within minutes or few hours of administration. One possible mechanism by which paraoxon administration improves survival of orally infected animals could be by increasing intestinal motility. This would lead to the rapid elimination of the bacteria from the intestinal lumen. However, when the intestinal motility was evaluated at 72 h after the last injection of paraoxon, a time at which mice were routinely infected in all of our studies, no enhancement in intestinal motility was detected. Furthermore, no difference was found in initial bacterial shedding between the experimental and control groups. Therefore, in our model, changes in intestinal motility are not the underlying cause of enhanced survival. We also investigated the possibility of paraoxon treatment inducing the release of secretory IgA, which is produced by plasma cells in the lamina propria and transported across epithelial cells to the intestinal mucosa. Previous studies have shown that ACh has a stimulatory effect on IgA secretion (53) but we were not able to detect any changes in the production of anti-*Salmonella* IgA or total IgA, either before or after infection.

Following oral inoculation with SL1344, differences in bacterial burden between control and experimental groups was observed as early as 4 days postinfection. At 7 days postinfection, bacterial load in paraoxon-treated group was significantly lower than that in the saline-treated group, both at mucosal (MLNs and feces) as well as systemic sites (spleen and liver). This was accompanied by decreased splenomegaly, and inflammatory cytokine production by spleen cells, and lower expression of activation markers in spleen and MLN cells. In this pathogenic *Salmonella* model, bacteria rapidly spread from the gut to organs like liver and spleen and mice die within several days (54). The fact that we were not able to detect an increase in the inflammatory response in the paraoxon-treated group, suggested that the lower bacterial load was not due to a stronger immune response in bacteria-clearing organs like the spleen. Instead, our data strongly suggest that pretreatment with AChE inhibitor was able to control the systemic dissemination of bacteria. Bioluminescence *in vivo* imaging of mice receiving a relatively high dose of virulent *Salmonella* indicated that bacteria were held in the abdominal area, most probably at the intestinal mucosal barrier. The delayed dissemination could be due to a lower number of bacteria able to invade the intestinal mucosa and a more effective mucosal antibacterial immune response.

The vagus nerve is the main cholinergic parasympathetic nerve that innervates the muscularis of the gastrointestinal tract (55). Within the intestinal submucosa, the submucous plexus of the enteric nervous system directly controls the intestinal epithelial and immune cell function through the release of ACh (56). Therefore, paraoxon-induced cholinergic stimulation could directly (*via* the vagus nerve) and/or indirectly (submucosal plexus) increase the levels of ACh in the submucosa and modulate the activity of the epithelial cells involved in antimicrobial defense (57). The intestinal epithelium constitutes an important part of the innate immune defense mechanism by contributing to the intestinal barrier that controls and restricts the entry of luminal pathogens. Two essential components of this barrier are the GC and PC. GC contribute to the production of a viscous substance composed of mucin-glycoproteins. Mucus is an important component of the first-line defense to oral pathogens like *Salmonella*. The higher concentration of mucus forms a layer that entraps oral pathogens, making it harder for *Salmonella* organisms to adhere to the epithelial cells and, therefore, penetrate and invade the intestinal epithelium. In our study, GC degranulation and the release of mucus were observed by EM and immunohistochemistry analysis in paraoxon-treated mice. This could limit the translocation of *Salmonella* organisms across the intestinal mucosa, providing the enhanced protection against infection. The fact that we could also detect, at early time points, a higher number of bacteria in the intestinal lumen of paraoxon-treated group highlights the essential role that the mucus barrier plays in halting bacterial translocation. In line with our results, early studies described that cholinergic stimulation induces mucus secretion in the intestine (16) and, moreover, this secretion was blocked by atropine, a muscarinic receptor antagonist, indicating that the ACh muscarinic receptors are involved in the induction of the mucus secretion (58).

Paneth cells are highly specialized epithelial cells located at the base of the Lieberkuhn’s crypts in the small intestine and are critical source of AMPs, which are released upon interaction with pathogens (59). Transgenic mice overexpressing a human defensin were more resistant to an oral, but not intraperitoneal, infection by virulent *Salmonella* by preventing pathogen invasion at the intestinal mucosa (60). Conversely, mice deficient in alpha-defensins (cryptdins) were more susceptible to oral infection by virulent *Salmonella* (61). Activation of the bactericidal activity of alpha-defensins, which are secreted by PC, requires the matrix metalloproteinase (MMP-7 or matrilysin), an enzyme that cleaves cryptdin precursors (61). Two additional molecules important for the innate host defense, Ang-4 and RegIIIγ, are also produced by PC in response to the presence of microbes in the intestinal lumen (62–64) and have been shown to be bactericidal to enteric pathogens, including *Salmonella* (65, 66). Our analysis of gene expression of different AMPs in ileal epithelial cells revealed that paraoxon treatment strongly upregulated the expression of RegIIIγ, which plays an important role in defense against Gram-positive and Gram-negative pathogens and in regulating the distribution of mucus in the ilium (66). Our findings indicate that following oral infection with *Salmonella*, saline-pretreated mice strongly upregulated the expression of RegIIIγ, Defa1, MMP-7, and Ang-4. This is most likely due to the interaction of PC with *Salmonella* organisms and serves to eliminate and/or prevent pathogen invasion. Surprisingly, the extent of upregulation of all four AMPs was lower in infected paraoxon-pretreated mice, which could be a consequence of a decreased exposure of PC to bacteria in the intestinal lumen. Interestingly, ultrastructural analysis of the small intestine showed that cholinergic stimulation induced the degranulation of PC in uninfected mice, causing the release of AMPs to the intestinal lumen. Several studies described the enhanced PC secretion by several cholinergic agonists (67, 68) and inhibition of their degranulation by atropine (17). Further studies demonstrated that bethanechol, a muscarinic agonist, induced PC secretion in the mouse intestine by direct interaction with the muscarinic receptors expressed on PC, indicating that ACh has a regulatory role in the degranulation of AMPs-containing granules (69). Taken together, these findings support our hypothesis that cholinergic stimulation enhances the mucosal innate immune defense in the intestinal tract against oral *Salmonella* infection.

We postulate that the degranulation of PC and the release of their AMPs could lead to a more efficient elimination of *Salmonella* organisms reaching the intestinal crypts. It is also known that degranulation and secretion of the content of PC induce their death (70). This supports our observations of disintegrated PC adjoining younger, newly generated, ones. PC can also respond to the secretion of pro-inflammatory cytokines released by T cells (71). A recent study in primary epithelial organoid cultures demonstrated that PC degranulation can also be induced by IFNγ (70). Our group has recently demonstrated that paraoxon treatment induces an increase in IFNγ-producing T cells (24). So, a question that remains to be answered is whether AChE inhibition induces the secretion of IFNγ by mucosal lymphocytes that could additionally stimulate PC degranulation. The findings of our study suggest the intriguing possibility that increased mucin production may act to limit the exposure of PC in the intestinal crypts to the lumen bacteria in paraoxon-pretreated mice, which could explain the lower level of AMPs induction observed in this group of animals.

In summary, our findings demonstrate the capacity of cholinergic pathway activation to induce protection against virulent oral enteric pathogens. This activity is associated with enhanced degranulation of GC and PC and the release of mucins and AMPs that, in turn, act to prevent bacterial attachment and invasion, and hence enhancing their elimination at the intestinal epithelium. This study offers a new perspective in the relationship between nervous and immune system that can be of enormous importance in innate immunity against oral pathogens. Our data suggest a possible neuroimmune modulation that operates by enhancing the gastrointestinal barrier defense mechanisms and leads to improved host survival after a lethal oral infection.

## Ethics Statement

This study was carried out in accordance with the recommendations of the Animal Research Ethics Committee of the College of Medicine and Health Sciences (CMHS). The protocol was approved by the Animal Research Ethics Committee (Protocol no.AE/06/81).

## Author Contributions

RA-B performed experiments and analyzed data. GB performed histological experiments and analyzed data. MQ performed biochemical assays for AChE activity and sample processing for EM studies. YM provided valuable support for all molecular studies. AA-S contributed to the flowcytometric analysis. ST performed EM studies. WL supervised and analyzed intestinal transit experiments. Ba-R designed the study, analyzed data, and wrote the final manuscript. MF-C designed the study, supervised the project, analyzed data, and wrote the final manuscript.

## Conflict of Interest Statement

The authors declare that the research was conducted in the absence of any commercial or financial relationships that could be construed as a potential conflict of interest. The reviewer SC and handling Editor declared their shared affiliation.
