# Supplementary material for: Cholinergic Activation Enhances Resistance to Oral Salmonella Infection by Modulating Innate Immune Defense Mechanisms at the Intestinal Barrier
Source: Front Immunol. 2018 Mar 19;9:551. doi: 10.3389/fimmu.2018.00551 (PMC5867304; doi:10.3389/fimmu.2018.00551)
Supplement: Supplementary file 1 [file Data_Sheet_1.PDF]

Cholinergic activation enhances resistance to oral Salmonella infection by modulating innate immune defense mechanisms at the intestinal barrier

Ray Al-Barazie[1], Ghada Bashir[2], Mohammed M. Qureshi[1], Yassir A. Mohamed[2], Ashraf Al-Sbiei[2], Saeed Tariq[3], Wim Lammers[4], Basel K. al-Ramadi[2] and Maria J. Fernandez-Cabezudo[1]

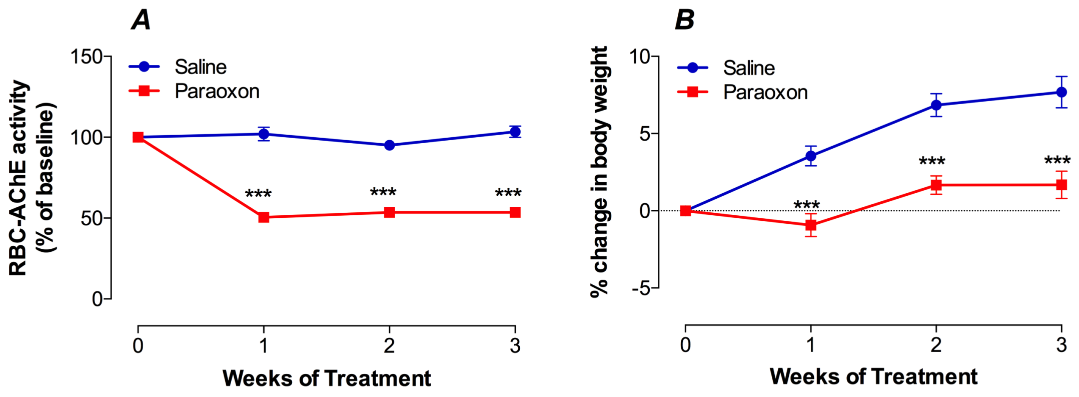

Supplementary Figure 1. Paraoxon treatment reduces RBC AChE activity and slows body weight growth. Mice were treated with paraoxon or saline for three weeks. (***A***) Blood was collected at the end of each week and enzyme activity was measured following modified Ellman's method. All enzyme activities were expressed in percentage of the baseline activity (100%). (***B***) Weekly change in body weight was calculated as percentage of the initial weight. All graphs represent the mean values ± SEM of cumulative data from 3-4 independent experiments. Asterisks denote statistically significant differences between the control and experimental groups (***$p<0.001$).

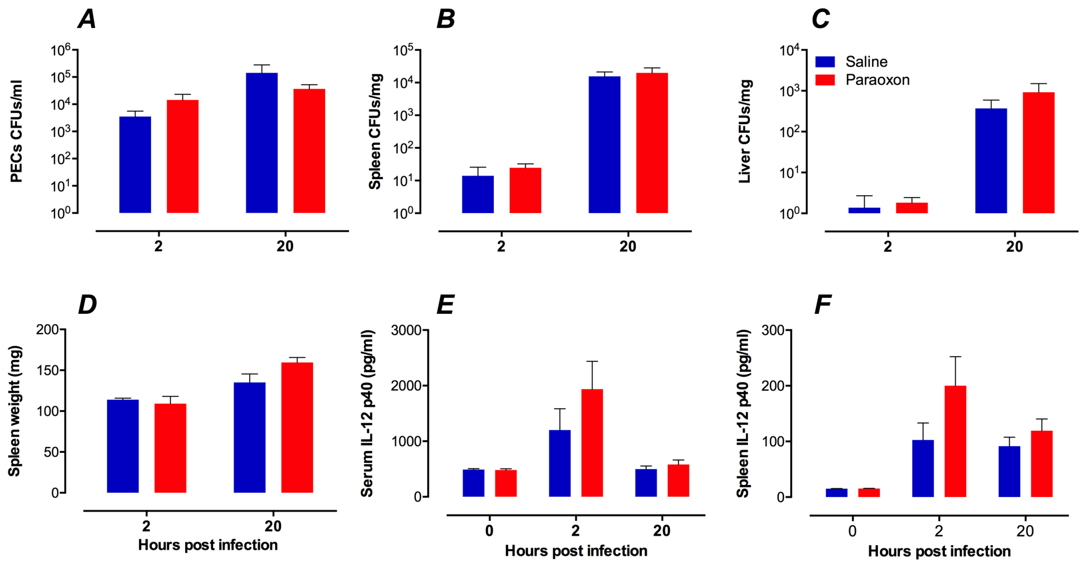

Supplementary Figure 2. Effects of cholinergic stimulation on systemic infection with SL1344. Mice treated with paraoxon or saline were infected i.p. with virulent Salmonella (strain SL1344; 1.2x10^3/mouse), and then sacrificed at the indicated time points. (***A***) Peritoneal fluid was harvested and bacterial loads determined as CFUs/ml. (***B***) Excised spleen and (***C***) liver were homogenized and bacterial loads were determined and calculated as CFUs/mg. (***D)*** Spleen weights were also analyzed. (***E***) IL-12p40 content in serum and (***F***) splenocyte 24h-culture-supernatant was assayed by ELISA. Values represent the mean ± SEM and are representative of 3 independent experiments.

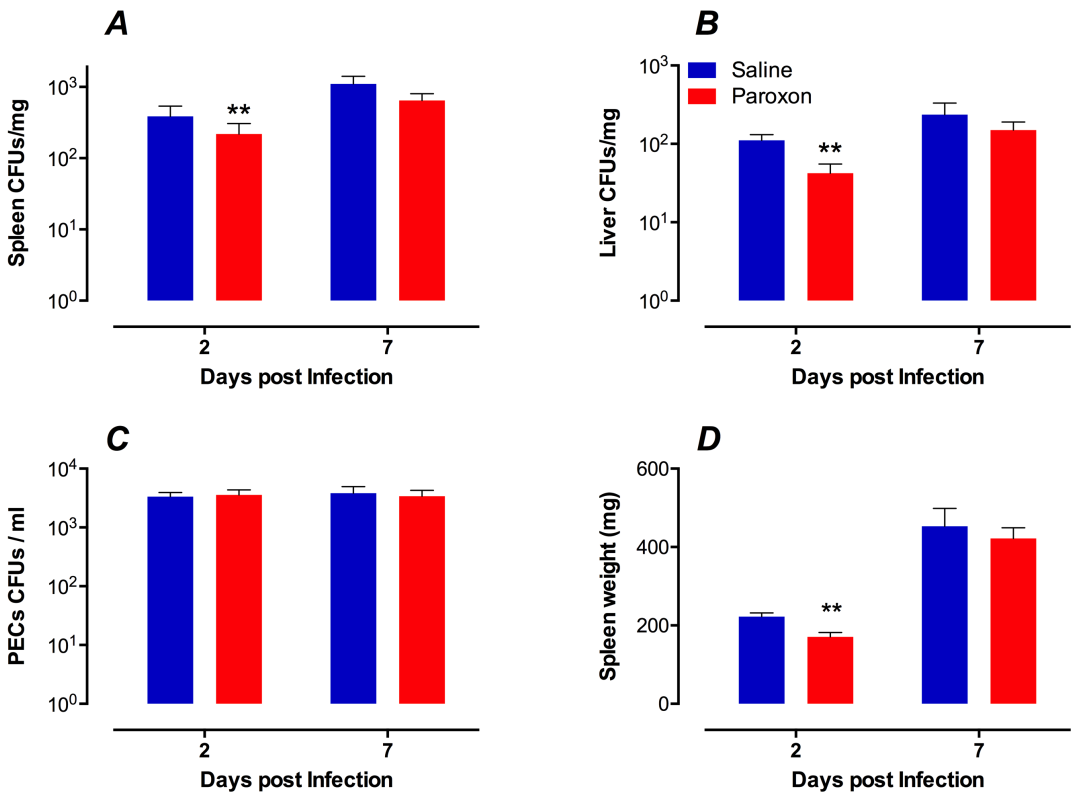

Supplementary Figure 3. Bacterial load in PECs and target organs after systemic infection with BRD509E. Mice pre-treated with paraoxon or saline were infected i.p. with attenuated Salmonella (strain BRD509E; 2-5x10^5/mouse) and sacrificed at days 2 or 7 post-infection. (***A***) Peritoneal fluid was retrieved and bacterial load determined as CFUs/ml. Bacterial load was also determined in (***B***) liver and (***C***) spleen as CFUs/mg. Figure (***D***) depicts spleen weights at the same time points. Each data point represents the mean ± SEM of cumulative data from 3-5 independent experiments. Asterisks denote statistically significant differences between control and experimental group at each time point (**$p \leq 0.01$).

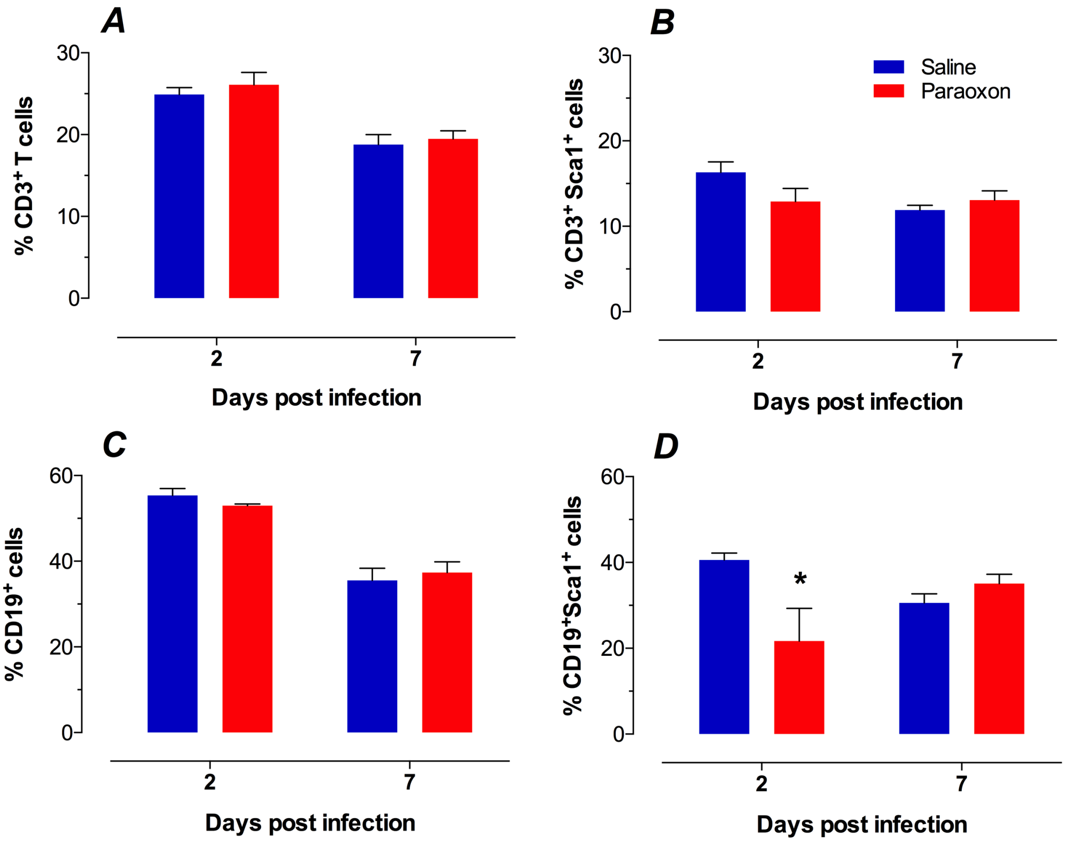

Supplementary Figure 4. Flowcytometric analysis of splenocytes after systemic infection with BRD509E. Mice were treated for three weeks with paraoxon or saline, and then infected i.p. with BRD509E ($2\text{-}5 \times 10^5$). Mice were sacrificed at the indicated time points. Erythrocytes-depleted splenocytes were analyzed by flowcytometry using specific mAbs. Graphs ***A-D*** depict percentages of (***A***) total T cells ($CD3^+$), (***B***) activated T cells ($CD3^+$/$Sca\text{-}1^+$), (***C***) total B cells ($CD19^+$) and (***D***) activated B cells ($CD19^+$/$Sca\text{-}1^+$). Data represent the mean values ± SEM from a representative of 2 independent experiments. Asterisks denote statistically significant differences between control and experimental group at each time point (* $p < 0.05$).

`

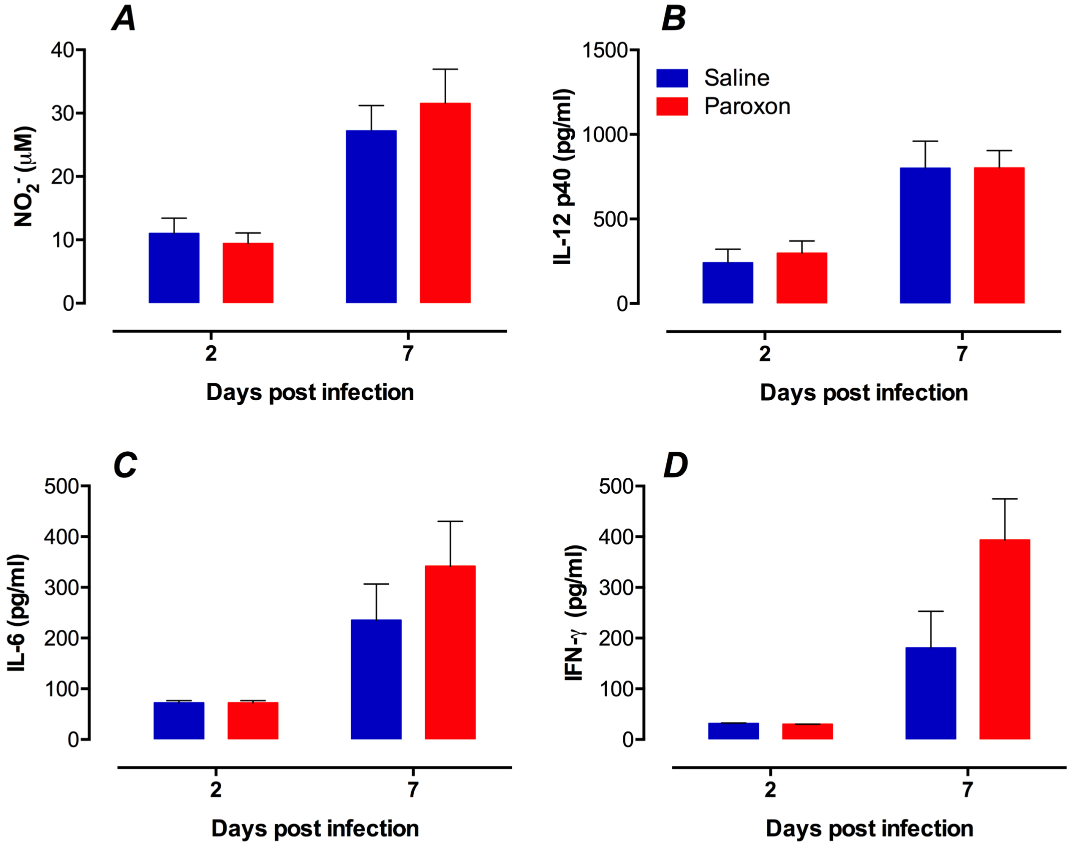

Supplementary Figure 5. Functional response of spleen cells following systemic infection with BRD509E. Following exposure to saline or paraoxon, mice were infected by i.p. administration of BRD509E (2-5x10^5) and sacrificed at day 2 or 7 post-infection. Spleen single cell suspensions were cultured, and 48-hour-culture supernatants analyzed for (***A***) nitrite content and (***D***) IFN-γ. 24 hour-supernatants were assayed for (***B***) IL-12p40 and (***C***) IL-6. Graphs depict mean ± SEM from 2-3 independent experiments.
